# A Web-Based Acceptance-Facilitating Intervention for Identifying Patients’ Acceptance, Uptake, and Adherence of Internet- and Mobile-Based Pain Interventions: Randomized Controlled Trial

**DOI:** 10.2196/jmir.9925

**Published:** 2018-08-21

**Authors:** Jiaxi Lin, Bianca Faust, David Daniel Ebert, Lena Krämer, Harald Baumeister

**Affiliations:** ^1^ Institute of Psychiatry, Psychology & Neuroscience Health Psychology Department King's College London London United Kingdom; ^2^ Institute of Sports and Sport Science Department of Sport Psychology University of Freiburg Freiburg Germany; ^3^ Rehabilitation-Center Todtmoos Clinic Wehrawald Todtmoos Germany; ^4^ Clinical Psychology and Psychotherapy Friedrich-Alexander University of Erlangen-Nürnberg Erlangen Germany; ^5^ Institute of Psychology Department of Rehabilitation Psychology and Psychotherapy University of Freiburg Freiburg Germany; ^6^ Institute of Psychology Department of Clinical Psychology and Psychotherapy University of Ulm Ulm Germany

**Keywords:** uptake, acceptance, adherence, eHealth, chronic pain, randomized controlled trial

## Abstract

**Background:**

Internet- and mobile-based interventions are effective for the treatment of chronic pain. However, little is known about patients’ willingness to engage with these types of interventions and how the uptake of such interventions can be improved.

**Objective:**

The aim of this study was to identify people’s acceptance, uptake, and adherence (primary outcomes) with regard to an internet- and mobile-based intervention for chronic pain and the influence of an information video as an acceptance-facilitating intervention (AFI).

**Methods:**

In this randomized controlled trial with a parallel design, we invited 489 individuals with chronic pain to participate in a Web-based survey assessing the acceptance of internet- and mobile-based interventions with the offer to receive an unguided internet- and mobile-based intervention for chronic pain after completion. Two versions of the Web-based survey (with and without AFI) were randomly sent to two groups: one with AFI (n=245) and one without AFI (n=244). Participants who completed the Web-based survey with or without AFI entered the intervention group or the control group, respectively. In the survey, the individuals’ acceptance of pain interventions, measured with a 4-item scale (sum score ranging from 4 to 20), predictors of acceptance, sociodemographic and pain-related variables, and physical and emotional functioning were assessed. Uptake rates (log in to the intervention) and adherence (number of completed modules) to the intervention was assessed 4 months after intervention access. To examine which factors influence acceptance, uptake rate, and adherence in the internet- and mobile-based interventions, we conducted additional exploratory subgroup analyses.

**Results:**

In total, 57 (intervention group) and 58 (control group) participants in each group completed the survey and were included in the analyses. The groups did not differ with regard to acceptance, uptake rate, or adherence (*P*=.64, *P*=.56, *P*=.75, respectively). Most participants reported moderate (68/115, 59.1%) to high (36/115, 31.3%) acceptance, with 9.6% (11/115) showing low acceptance (intervention group: mean 13.91, SD 3.47; control group: mean 13.61, SD 3.50). Further, 67% (38/57, intervention group) and 62% (36/58, control group) had logged into the intervention. In both groups, an average of 1.04 (SD 1.51) and 1.14 (SD 1.90) modules were completed, respectively.

**Conclusions:**

The informational video was not effective with regard to acceptance, uptake rate, or adherence. Despite the high acceptance, the uptake rate was only moderate and adherence was remarkably low. This study shows that acceptance can be much higher in a sample participating in an internet- and mobile-based intervention efficacy trial than in the target population in routine health care settings. Thus, future research should focus not only on acceptance and uptake facilitating interventions but also on ways to influence adherence. Further research should be conducted within routine health care settings with more representative samples of the target population.

**Trial Registration:**

German Clinical Trial Registration DRKS00006183; http://www.drks.de/drks_web/navigate.do ?navigationId=trial.HTML&TRIAL_ID=DRKS00006183 (Archived by WebCite at http://www.webcitation.org/70ebHDhne)

## Introduction

Chronic pain as a disease in its own right is a major global health problem [1-3]. In the Global Burden of Disease Study of 2013 [4], low back pain, neck pain, and migraine, which often take a chronic course, were found among the top 10 causes of years lived with disability in every country under investigation. This not only reflects the high prevalence of chronic pain affecting 1 in 5 adults [2,5] but also the urgent need to improve health care. A large-scale survey of chronic pain in Europe found that 40% of the participants reported that their pain was inadequately controlled and only 2% were treated by pain specialists [2].

Therefore, innovative, effective, and cost-effective health care models for chronic pain are needed. This should include a multimodal, biopsychosocial approach, considered as the gold standard in the treatment of pain [6-8], with self-management where possible. In this context, internet- and mobile-based interventions (IMIs) might be a feasible means to provide psychological interventions such as cognitive behavioral interventions [9-14]. As most IMIs provide evidence-based strategies as interactive self-help lessons on a Web-based platform, they can reach large numbers of people simultaneously, anytime and anywhere [15-17]. A rising number of studies indicate the efficacy of IMIs for a wide range of mental and physical health conditions including chronic pain, depression, and anxiety [16-21]. A recent meta-analysis by Buhrman et al [9] on IMIs for chronic pain found small but significant positive effects for interference or disability, pain intensity, catastrophizing, and depression at Hedge’s g=−0.39, g=−0.33, g=−0.49, and g=−0.26, respectively. A recent randomized controlled trial (RCT) on an IMI based on Acceptance and Commitment Therapy (ACT) showed guided, but not unguided, IMIs being effective in improving pain interference (Cohen *d*=0.58 at posttreatment and follow-up, respectively [22]) in individuals with chronic, nonmalignant pain for 6 months or longer. Moreover, changes in psychological flexibility mediated all outcomes of ACT-based online treatment for chronic pain (ACTonPain) [23], and cost-effectiveness analyses revealed that ACTonPain is potentially cost-effective, depending on the amount of society’s willingness to pay [24]. In this trial, the uptake rate was 97% in both ACTonPain groups, and guided participants completed more modules (0-8) than those who were unguided (mean 5.94, SD 2.80 vs mean 4.74, SD 2.89, *F*_1,199_=8.92; *P*<.01). The overall effect sizes in pain IMIs are in line with the effects of psychological interventions in face-to-face settings [25]. Hence, IMIs have the potential to improve chronic pain health care by providing evidence-based, possibly cost-effective psychological interventions [9,16-25] with high accessibility and scalability [15-17].

Two main barriers have repeatedly been discussed to limit the full potential of IMIs when implemented in routine health care settings: low uptake rates (logging into the intervention) and low levels of adherence (completing modules of the intervention) [26,27]. Evidence from multiple pragmatic studies examining depression IMIs implemented in real-life settings under less-structured and monitored conditions indicates that uptake rates vary between 3% and 25% [28-31]. Low intervention adherence in IMIs is a frequently reported problem as it can ultimately limit the effectiveness of IMIs [26,32-35]. In an RCT on the effectiveness of an ACT-based IMI for chronic pain, Trompetter et al [36] found that participants in the intervention and waitlist control group differed in pain interference only in the analysis with treatment completers. In routine health care settings, in contrast to developer-led efficacy trials on the same IMI [37-39], the issue of adherence seems to be particularly important when IMIs are offered.

A repeatedly suggested reason for low uptake and adherence is the low level of patients' acceptance of IMIs, conceptualized as the intention to use the intervention [40-42]. Other factors, such as internet usage and anxiety [41,43], uncertainty concerning data security, discomfort with use of IMIs and psychological interventions in general, and social influence by friends, family, and health professionals as well as a lack of trust in the effectiveness of IMIs are often reported to influence the acceptance and uptake of IMIs [40,42,44-47].

Aiming at these aspects of acceptance, acceptance-facilitating interventions (AFIs) are suggested to reduce patients´ apprehensions and misconceptions about IMIs. They provide trustworthy information on, as well as an introduction to IMIs [40,48-51]. To date, 3 RCTs have investigated the influence of a video-based [42,47] or personal [46] AFI in the clinical population of pain [47], diabetes [46], and primary care patients with depressive symptoms [42]. All studies consistently reported low baseline acceptance and an increase in acceptance following AFI [42,46,47,52]. However, all three studies only examined patients’ acceptance and lack more important information on whether AFI effectively increased intervention uptake.

Only two studies have reported on the relationship between IMI acceptance and IMI usage [27,53]. In both studies, a significant association was found between IMI acceptance and usage (log-in and adherence). This finding suggests that AFIs might also influence IMI usage. However, research on the influence of an AFI on intervention uptake and adherence is missing.

Therefore, in this study we examined whether an informational video (AFI) can increase patients’ (1) acceptance of an IMI for chronic pain, (2) uptake of an IMI for chronic pain, and (3) adherence in an IMI for chronic pain.

We expected that AFI would positively increase patients’ acceptance as well as the uptake rate and adherence. In addition, we expected that AFI would increase the predictors of acceptance and have a reducing effect on internet anxiety. To examine which factors influence acceptance, uptake rate, and adherence in IMIs, we conducted additional exploratory subgroup analyses.

## Methods

### Study Design

This study is linked to an outcome evaluation study with the German Clinical Trial Registration (DRKS): DRKS00006183, which is approved by the Ethics Committee of the Albert-Ludwigs-University of Freiburg. This trial is reported in accordance with the Consolidated Standards of Reporting Trials of Electronic and Mobile HEalth Applications and onLine TeleHealth checklist [54]. This was a two-arm pragmatic study using a parallel-group design with balanced (1:1) randomization. The intervention group (IG) received AFI with a subsequent Web-based survey (homepage provided by the University of Freiburg, Germany); the control group (CG) filled out the same Web-based survey without receiving AFI. In this RCT, randomization took place before the assessment of eligibility and inclusion of participants. We chose this procedure as it allowed us to send an invitation email providing a link to the survey in either the IG or CG condition. This is a case of randomization before data are available to confirm the individuals’ eligibility without risking bias in the analysis [55]. Therefore, postrandomization exclusions of all noneligible participants can be regarded as acceptable [55].

Reading and providing online informed consent and answering the survey took about 20-30 minutes. After completing the survey, the participants could choose to receive the unguided version of ACTonPain [22,56] by providing their email address in order to access ACTonPain.

### Sample

The recruitment took place in September 2015. We sent email invitations to all individuals to participate in this study who had earlier expressed interest in participating in an evaluation study on ACTonPain [22,56]. Individuals in the following categories could not be included in the evaluation study on ACTonPain for the following reasons: (1) screening or baseline assessment not completed or no informed consent for main trial (n=332) or (2) expressed their interest in participating after the target sample size of the main trial was reached (n=157). Applicants for participation in the main trial indicating an elevated risk of suicide were not invited. We assessed the following inclusion criteria based on the Web-based self-report: (1) ≥18 years of age, (2) pain duration ≥3 months, (3) sufficient German language skills, and (4) sufficient computer and internet skills to proceed with the Web-based questionnaire. We excluded all participants with an incomplete informed consent form and those not fulfilling the inclusion criteria. The intervention ACTonPain was conceptualized for chronic pain as a disease in its own right and not as a symptom of any specific disease (eg, chronic low back pain, migraine, or fibromyalgia) [1,56]. Moreover, ACT is applicable as a general therapeutic model [57], and therefore, no further specification concerning any specific disease related to chronic pain was made. All participants had full access to treatment as usual.

This study aimed at a minimal sample size of 102 participants to detect a clinically relevant medium effect size (Cohen *d*=0.50) with a power of 80% and a two-sided 5% significance level.

### Randomization and Allocation

**Figure 1:**
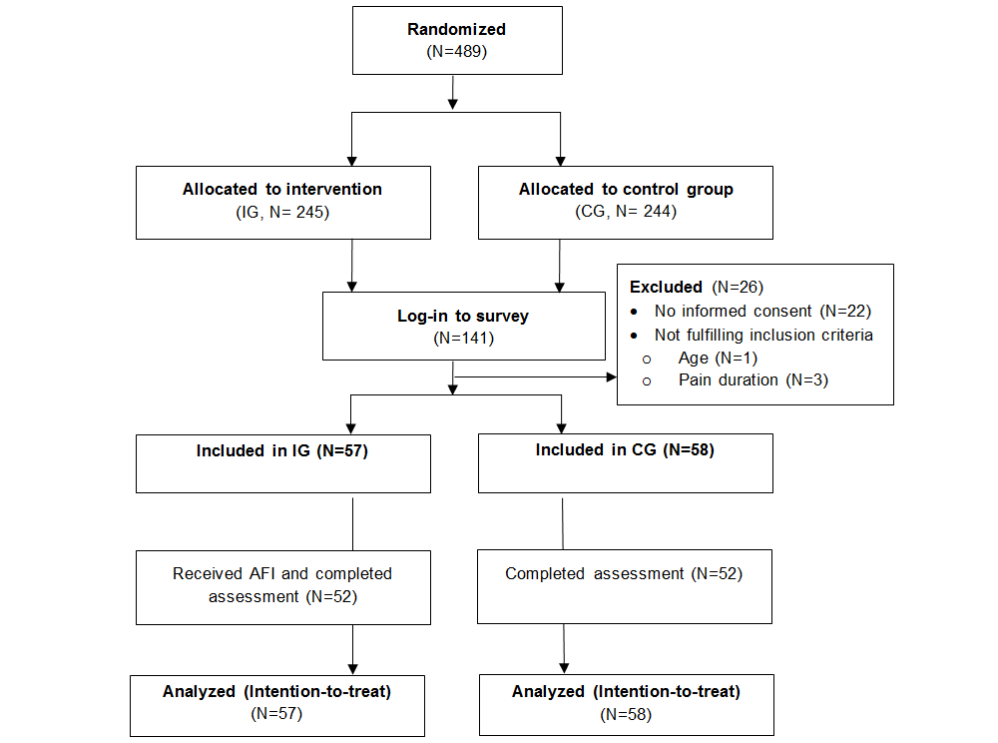
Flow chart. AFI: acceptance-facilitating intervention; CG: control group; IG: intervention group.

For allocation to IG or CG, a computer-generated list of random numbers with randomly varying block sizes of 4, 6, and 8 was used by BF (the sealedenvelope website). IG watched an AFI video before answering the Web-based questionnaire. CG filled out the questionnaire immediately. Out of 489 potential participants, 115 provided informed consent and fulfilled the inclusion criteria (Figure 1).

### Sociodemographic Data, Clinical Characteristics, and Internet Usage

The questionnaire comprised sociodemographic items concerning age, sex, relationship status, education, and employment status. Moreover, we asked participants about current or past psychological pain treatment (yes or no) and how satisfied they were with it.

#### Pain

Participants evaluated their actual pain as well as the worst, least, and average pain during the last week on a scale from 0 to 10. Additionally, pain duration was assessed with 5 categories ranging from “1 month to 6 months” to “>5 years.”

#### Physical Functioning

The Interference Scale of the Multidimensional Pain Inventory (MPI [58,59]) was used to measure the degree of pain interference with regard to all-day activities. This questionnaire is a valid measure of the interference of pain with physical functioning [60]. The Cronbach alpha in this study was at .91.

#### Emotional Functioning

We used the Patient Health Questionnaire depression scale (PHQ-8 [61-64]) for depressive symptomatology and the Generalized Anxiety Disorder Screener 7-item (GAD-7 [65]) for symptomatology associated with generalized anxiety disorder.

PHQ-8 assesses all Diagnostic and Statistical Manual of Mental Disorders, fifth edition (DSM-V) symptoms of major depression with the exception of suicidal or self-injurious thoughts during the last 2 weeks. Ratings are given on a 4-point Likert scale ranging from 0 ‘‘not at all’’ to 3 ‘‘nearly every day.’’ The scores for each item are summed up to produce a total score between 0 and 24 points. A cutoff score of 5-9 represents mild depressive symptoms; 10-14, moderate; 15-19, moderately severe; and 20-24, severe [62]. The Cronbach alpha of PHQ-8 was at .81 in this study.

GAD-7 consists of 7 core symptoms of the DSM-V diagnostic criteria A, B, and C for generalized anxiety [65]. The items are scored from 0 “not at all” to 3 “more than half the days” regarding the last 2 weeks. Scores range from 0 to 21; the cutoff points of 5, 10, and 15 represent the thresholds for mild, moderate, and severe anxiety symptom levels, respectively [65]. The Cronbach alpha in this study was at .87.

#### Internet Usage

We measured internet usage using the question ‘‘How often do you surf the internet?’’ Answers are rated on a 5-point Likert scale ranging from 1 ‘‘seldom or never’’ to 5 ‘‘multiple times per day.”

### Acceptance-Facilitating Intervention

**Figure 2:**
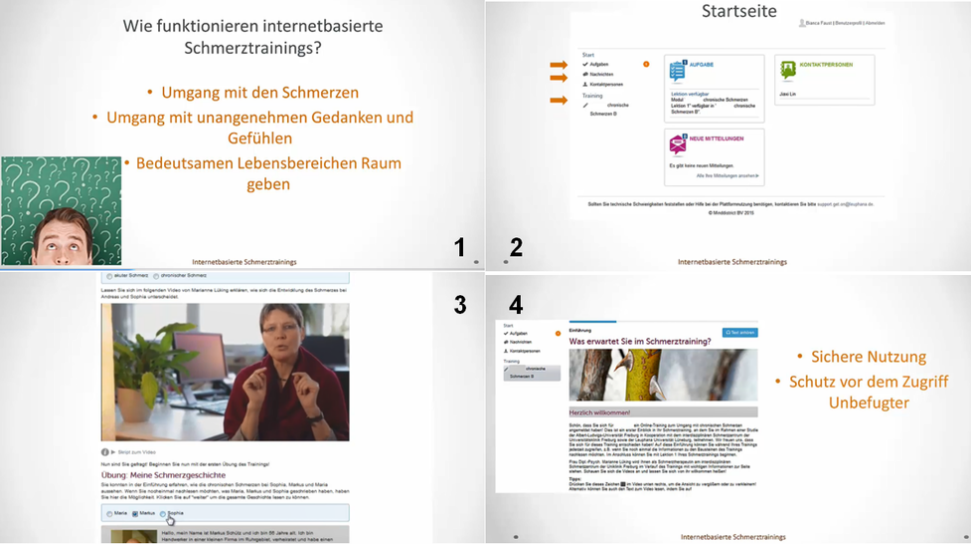
Screenshots of the acceptance-facilitating intervention. (1) content of an online pain intervention; (2) introduction to Acceptance and Commitment–based online treatment for chronic pain (ACTonPain) log-in page; (3) introduction to ACTonPain features; and (4) information concerning data security.

AFI consisted of a 3-minute introductory and information video to ACTonPain with screenshots of the program in order to improve patients’ acceptance. Figure 2 provides screenshots of AFI. We designed the content of the intervention to address the aforementioned barriers and drivers of acceptance. We conceptualized the video based on our previous AFIs that showed to be effective in increasing acceptance [42,47,66]. Our AFI is an adopted version of AFI used in a former study with individuals with chronic pain [47] with a specific introduction to ACTonPain. The content of the video comprised information on (1) the effectiveness of IMIs, (2) data security and anonymity in IMIs, (3) various advantages of IMIs (eg, ease and comfort of use, flexible time management), (4) the possibility of receiving technical support, and (5) assistance during the program. Furthermore, the video presented the process for using ACTonPain, encompassing the log-in or log-off processes and an overview of the modules and different features (audio files, video clips, and homework assignments).

### Acceptance and Commitment-Based Online Treatment for Chronic Pain

After completing the questionnaire, participants were invited to receive ACTonPain treatment in an unguided version and without short message service (SMS) text messages (SMS Coach). This version of ACTonPain was provided without any human support and should be therefore of special interest for public health services due to its high scalability and low costs.

ACTonPain consists of an introduction and 7 consecutive modules. The intervention targets core change processes proposed by Hayes et al [57] and is described in more detail by Lin et al [56]. Participants were advised to complete one session per week with a completion time of approximately 60 minutes. Participants could access ACTonPain via the Web or on their mobile phone with an adapted mobile view. ACTonPain was not delivered as a mobile phone app, and AFI demonstrated the use of ACTonPain via the Web.

The effectiveness of ACTonPain was investigated in a three-armed RCT with a total of 302 participants who were randomly assigned to either ACTonPain guided, ACTonPain unguided, or waitlist control. Guidance was given by trained eCoaches (psychologists) who provided individualized standardized feedback after each module and reminded the participants to keep to the schedule of the treatment and set up deadlines. Additionally, participants could receive supportive SMS text messages (SMS Coach).

### Measures

#### Primary Outcomes

The primary outcomes were acceptance, uptake, and adherence.

##### Acceptance

We operationalized acceptance on the basis of the well-established unified theory of acceptance and use of technology (UTAUT [67,68]). This framework provides a reliable theoretical basis of drivers and barriers for users’ acceptance of information technology [67-69] and has been used in numerous IMIs studies [27,47,66,70-73]. The UTAUT model postulates acceptance as the intention to use technology and the proximal predictor for actual use [74].

The items of the UTAUT acceptance were developed based on previous studies [46,47]. The sum score of the scale ranges from 4 to 20, and the 3 levels of acceptance can be categorized: low (sum score: 4-9), moderate (sum score: 10-15), and high (sum score: 16-20). The Cronbach alpha in this study was relatively low at .71. Table 1 provides an overview of the items for acceptance and predictors of acceptance (see secondary outcomes) in this study, including their scales.

##### Uptake

We operationalized uptake as log-in (yes or no) to IMI assessed 4 months after intervention access. The period of 4 months was chosen, as this should have been enough time for the participants to start with the intervention and work through all 8 modules. We assumed that 4 months after intervention access is a reasonable time to assess uptake and adherence.

##### Adherence

We operationalized adherence as the number of completed modules of the intervention assessed 4 months after intervention access.

#### Secondary Outcomes

The secondary outcomes were the predictors of acceptance according to UTAUT as well as internet anxiety.

##### Predictors of Acceptance

According to the UTAUT model, there are 4 key predictors of either the behavioral intention or usage behavior of IT: performance expectancy, effort expectancy, social influence, and facilitating conditions [68]. The items measuring the construct’s performance expectancy and effort expectancy were drawn from Vance et al [75]. The items for social influence and facilitating conditions were adapted from Venkatesh et al [68].

**Table 1:** Items of acceptance and predictors of acceptance according to the unified theory of acceptance and use of technology model.

Outcomes			Items	Rating scale		Reliability	
Acceptance			If offered, I intend to try out an internet-based psychological pain intervention If offered, I intend to use an internet-based psychological pain intervention regularly I would recommend an internet-based psychological pain intervention to a friend I am willing to pay for an internet-based psychological pain intervention	5-point scale (1 “does not apply at all” to 5 “applies completely”)		.71	
**Predictors of acceptance**							
	Performance expectancy	Using an internet-based psychological pain intervention would increase the effectiveness of my pain treatment Using an internet-based psychological pain intervention would be beneficial for my health care Overall, an internet-based psychological pain intervention would support me in coping with my chronic pain			5-point scale (1 “does not apply at all” to 5 “applies completely”)		.86
	Effort expectancy	Using an internet-based psychological pain intervention would be simple Using an internet-based psychological pain intervention would be an easy task for me An internet-based psychological pain intervention would be clear and easily comprehensible to me			5-point scale (1 “does not apply at all” to 5 “applies completely”)		.79
	Social influence	People close to me would recommend me to use an internet-based psychological pain intervention My general practitioner would recommend me to use an internet-based psychological pain intervention			5-point scale (1 “does not apply at all” to 5 “applies completely”)		.69
	Facilitating conditions	I do have all necessary technical preconditions for using an internet-based psychological pain intervention In case of technical problems with an internet-based psychological pain intervention, I would receive technical support			5-point scale (1 “does not apply at all” to 5 “applies completely”)		Two separate items, not a uniform scale

##### Internet Anxiety

Two items for internet anxiety were adapted from Venkatesh et al [68] (1) “The internet is something threatening to me” and (2) “I am afraid of making an irrevocable mistake while using the internet”). The items were rated on a 5-point Likert scale ranging from 1 “does not apply at all” to 5 “applies completely.” The Cronbach alpha in this study was at .69.

### Statistical Analyses

We conducted data analysis using SPSS Statistics 22 (IBM Corporation, Armonk, NY, USA). As only those participants who completed the Web-based survey were included in the analysis, there were no missing data in this study. Descriptive statistics are provided for sociodemographic data and functioning to describe the sample. To test for randomization imbalance between IG and CG, we employed chi-square tests and *t*-tests for independent samples. The descriptive statistics were based on nonimputed data, while all following analyses were conducted after multiple imputations with 20 imputations using the imputation algorithm implemented in SPSS (intention-to-treat analysis).

To detect differences between IG and CG regarding acceptance, uptake, adherence, and the predictors of acceptance as well as internet anxiety, we conducted *t*-tests for independent samples and chi-square tests. In case of significant group differences, standardized mean differences (Cohen *d*) with a 95% CI were computed to quantify the effect. As this study includes multiple primary outcomes, we used a Bonferroni adjustment for the *P* values of .02 (3 tests at an alpha level of .05). This procedure resulted in sufficient statistical power with the sample to detect differences between the two conditions that were larger than Cohen *d*=0.65.

To examine potential subgroup differences (age, gender, education, pain duration and intensity, prior or present psychological intervention, internet usage and anxiety, and physical and emotional functioning) regarding acceptance, uptake, and adherence, exploratory analyses are provided (mean, SD, *t*-tests, and chi-square test). For this purpose, variables were dichotomized using defined cutoffs (gender, pain duration, education, and psychological intervention) or a median split (age, pain intensity, internet usage and anxiety, physical and emotional functioning, and level of acceptance regarding uptake and adherence). Note that the results of the subgroup analyses and analysis on secondary outcomes are exploratory and underpowered; adjusting for multiple testing would not be meaningful [76].

## Results

### Participants

Of 489 persons, 141 (28.8%) responded to the invitation. After we excluded those who did not provide informed consent (n=22) or did not fulfill the inclusion criteria (n=4), we included 57 and 58 participants in IG and CG, respectively. The missing value was between 0% and 5.7% per variable, and Little’s Missing Completely at Random test indicated that the data were missing at random (χ^2^_41_=45.31, *P*=.30).

The majority (82/115, 71.3%) of the participants were female. Ages ranged from 18 to 76 years with a mean age of 50.42 (SD 13.67) years. The majority of the sample (96/115, 83.5%) reported a pain duration of longer than 2 years, with 57.4% (66/115) suffering for more than 5 years. In addition, 86.1% (99/115) and 75.7% (87/115) of the participants reported at least mild symptomatology of depression and anxiety, respectively. Table 2 shows sociodemographic and clinical characteristics as well as internet usage in the sample. No significant differences for demographic and pain- and function-related variables were found between the two groups except in regard to employment, as more participants in CG were employed. Differences between IG and CG in all outcomes are summarized in Table 3.

### Primary Outcomes

**Figure 3:**
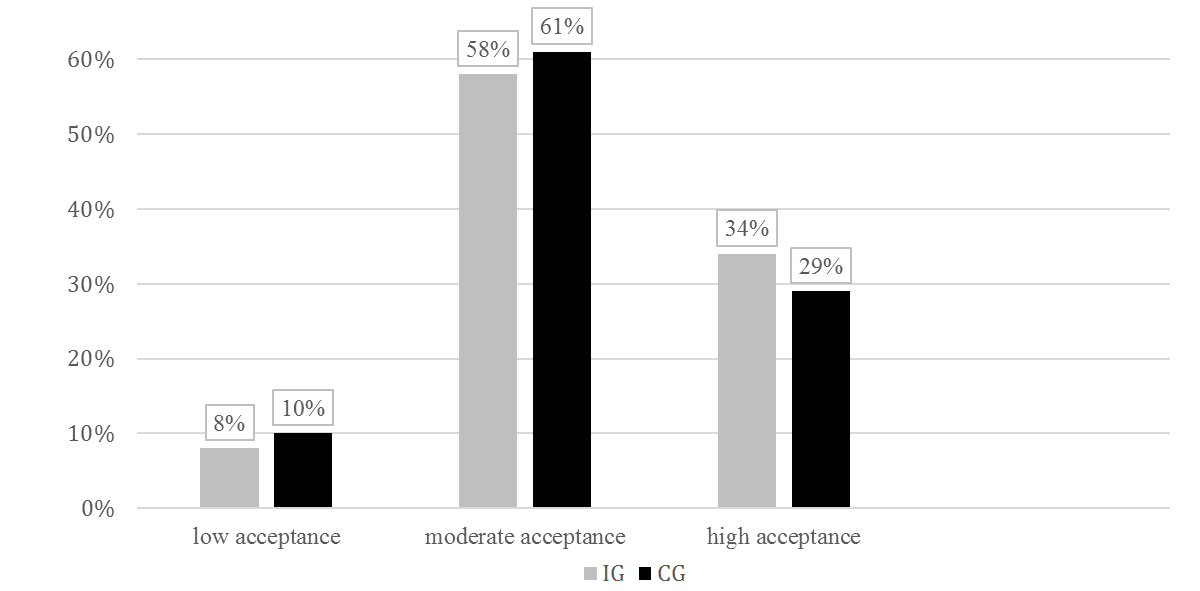
Level of acceptance. CG: control group; IG: intervention group.

There was no significant difference between IG and CG with regard to acceptance, uptake, or adherence (*P*=.64, *P*=.56, *P*=.75, respectively). Among the total sample, 8.7% (10/115) showed a low, 59.1% (68/115) a moderate, and 31.3% (36/115) a high level of acceptance, with an average sum score of 13.76 (SD 3.54). Figure 3 displays the levels of acceptance in both groups. The participants who applied for access to ACTonPain numbered 48 in IG and 50 in CG.

**Figure 4:**
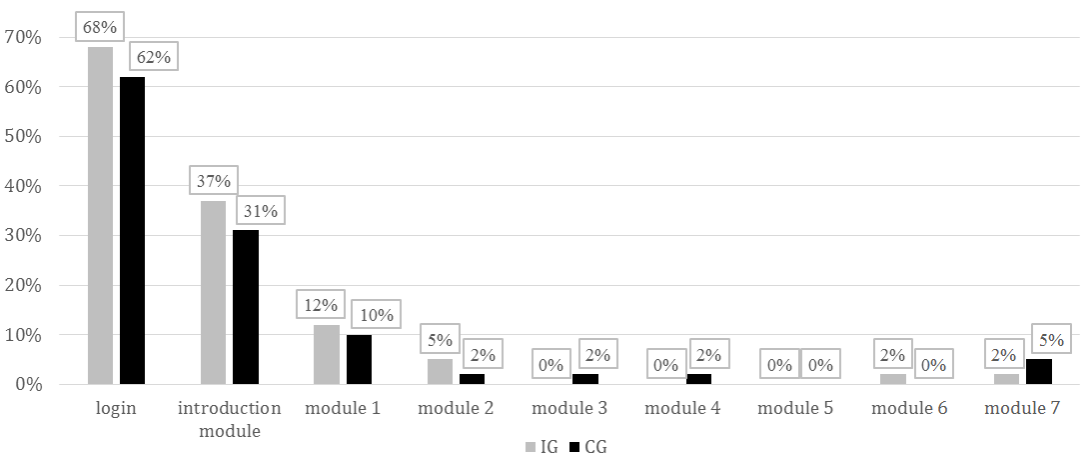
Number of log-ins and completed modules. CG: control group; IG: intervention group.

Note that 9% (5/57) and 10% (6/58) of participants in IG and CG, respectively, did not complete the survey and therefore did not indicate whether they wanted to receive the intervention. Then, 7% (4/57) and 3% (2/58) of participants in IG and CG, respectively, did not want to receive the intervention, and 84% (48/57) and 86% (50/58) of participants in IG and CG, respectively, signed up to receive the intervention. Four months after receiving access to ACTonPain, 65.2% (75/115) of the sample had logged in. This represents an uptake rate of 68% (38/57, IG) and 62% (36/58, CG). With regard to adherence, the participants completed 1.09 (SD 1.72) modules on average. That is, the average participant only completed the introduction module. The results showed that 5.2% (6/115) participants did not complete any modules after log-in and 3.5% (4/115) completed all the modules in the study. Hence, the treatment dropout rate was at 96.5% (111/115). Figure 4 presents the number of log-ins and completed modules in each group.

### Secondary Outcomes

There was no significant difference between IG and CG with regard to performance expectancy, effort expectancy, social influence, facilitating conditions, or internet anxiety (*P*=.88, *P*=.16, *P*=.96, *P*=.69, *P*=.68, respectively; Table 2).

### Subgroup Analyses

Since there were no group effects, we conducted the subgroup analyses with no group consideration in order to increase the power of the analyses. Participants with lower internet anxiety and higher anxiety symptoms showed significantly higher acceptance than their equivalent counterparts (Table 4). With regard to uptake rates, more participants with higher depressive symptoms (45/60, 75%) and acceptance (47/59, 80%) logged into the platform than those with lower depressive symptoms (30/55, 55%) and acceptance (28/56, 50%). We also found that participants with a higher level of acceptance completed more modules compared with participants with a lower level of acceptance (1.43 vs 0.72 modules).

**Table 2:** Sociodemographic and clinical characteristics and internet usage.

Characteristics			Total (N=115)		Intervention group (n=57)		Control group (n=58)		*P* value^a^	
**Sociodemographic characteristics**										
	Age (years), mean (SD)			50.42 (13.32)		51.65 (14.02)		49.21 (12.60)		.33
	Sex (female), n (%)			82 (71.3)		33 (57.9)		39 (67.2)		.53
	Married or in a relationship, n (%)			77 (66.9)		37 (64.9)		40 (69.0)		.69
**Educational level, n (%)^b^**										
	No school-leaving qualification			25 (21.7)		13 (22.8)		12 (20.7)		.82
	Lower secondary			10 (8.7)		7 (12.3)		3 (5.2)		.20
	Middle secondary			20 (17.4)		8 (14.0)		12 (20.7)		.46
	Higher secondary			6 (5.2)		2 (3.5)		4 (6.9)		.68
	Highest secondary			12 (10.4)		6 (10.5)		6 (10.3)		.99
	Vocational training			24 (20.9)		14 (24.6)		8 (13.8)		.16
	University degree			18 (15.6)		5 (8.8)		11 (19.0)		.18
**Employment**										
	(Self-) Employed, n (%)			63 (54.8)		24 (42.1)		39 (67.2)		.01
**Pain**										
	Intensity, mean (SD)			4.62 (1.72)		4.83 (1.35)		4.55 (1.88)		.46
	**Duration, n (%)**									
		3-6 months		2 (1.7)		1 (1.7)		1 (1.7)		.99
		1-2 years		17 (14.8)		6 (10.5)		11 (19.0)		.29
		2-5 years		30 (26.1)		17 (29.8)		13 (22.4)		.40
		Over 5 years		66 (57.4)		33 (57.9)		33 (56.9)		.99
	Prior psychological pain treatment^c^ (n=111), n (%)			49 (42.6)		22 (38.6)		27 (46.5)		.41
	Current psychological pain treatment^c^ (n=111), n (%)			24 (20.9)		13 (22.8)		11 (19.0)		.65
**Physical functioning**										
	Multidimensional Pain Inventory, mean (SD)			3.79 (1.09)		3.81 (1.04)		3.77 (1.34)		.84
**Emotional functioning (n=108)**										
	PHQ-8^d^, mean (SD)^c^			10.67 (4.86)		10.79 (4.79)		10.55 (4.97)		.79
	GAD-7^e^, mean (SD)^c^			8.23 (4.86)		8.42 (5.24)		8.04 (4.46)		.69
Internet usage, mean (SD)			3.43 (1.21)		3.21 (1.18)		3.64 (1.22)		.06	

^a^The *P* value refers to the significance level of the test on differences between the intervention and control groups on sociodemographic and clinical characteristics and internet usage.

^b^Secondary education according to the German classification: “Hauptschule” (“lower,” 9 years, until age 15/16), “Realschule” (“middle,” 10 years, until age 16/17), “Fachhochschulreife” (“higher,” 12 years, until age 17/18), “Abitur” (“highest,” 12 or 13 years, until age 17-19).

^c^Incomplete data.

^d^PHQ-8: Patient Health Questionnaire depression scale.

^e^GAD-7: Generalized Anxiety Disorder Screener 7-item.

**Table 3:** Differences between the intervention and control groups in all outcomes (intention-to-treat analysis dataset).

Outcomes		Total (N=115)	Intervention group (n=57)	Control group (n=58)	*P* value
**Primary outcomes**					
	Acceptance, mean (SD)	13.76 (3.54)	13.91 (3.47)	13.61 (3.50)	.64
	Uptake, n (%)	75 (65.2)	39 (68.4)	36 (62.1)	.56
	Adherence, mean (SD)	1.09 (1.72)	1.04 (1.51)	1.14 (1.90)	.75
**Secondary outcomes, mean (SD)**					
	Performance expectancy	9.82 (2.79)	9.78 (3.10)	9.86 (2.51)	.88
	Effort expectancy	10.85 (2.90)	10.47 (3.02)	11.23 (2.82)	.16
	Social influence	5.88 (2.36)	5.89 (2.42)	5.87 (2.36)	.96
	Facilitating conditions	7.45 (2.14)	7.53 (2.11)	7.37 (2.21)	.69
	Internet anxiety	3.15 (1.61)	3.21 (1.51)	3.09 (1.60)	.68

**Table 4:** Subgroup-specific effects on acceptance, uptake, and adherence, intention-to-treat analysis dataset.

Subgroups			Acceptance				Uptake rate				Adherence			
			Mean (SD)		*P* value		n (%)		*P* value		Mean (SD)		*P* value	
**Age**					.64				.33				.85	
	<51 (n=54)			13.92 (2.87)				38 (70)				1.06 (1.62)		
	≥51 (n=61)			13.62 (3.91)				37 (61)				1.11 (1.81)		
**Sex**					.44				.99				.89	
	Female (n=8)			13.93 (3.26)				53 (64)				1.07 (1.69)		
	Male (n=33)			13.35 (4.05)				22 (67)				1.12 (1.82)		
**Educational level**					.63				.44				.50	
	Low^a^ (n=60)			13.61 (2.98)				37 (62)				0.98 (1.71)		
	High^b^ (n=55)			13.93 (2.80)				38 (69)				1.20 (1.87)		
**Pain intensity**					.75				.17				.78	
	<4.50 (n=52)			13.65 (3.60)				30 (58)				1.04 (1.73)		
	≥4.50 (n=63)			13.85 (3.49)				45 (71)				1.13 (1.86)		
**Pain duration**					.52				.32					
	<5 years (n=49)			13.52 (3.57)				29 (59)				0.92 (1.60)		.37
	≥5 years (n=66)			13.94 (3.41)				46 (70)				1.21 (1.80)		
**Former psychological intervention**					.43				.99				.77	
	Ever (n=52)			13.59 (3.97)				34 (65)				1.18 (1.80)		
	Never (n=63)			14.11 (2.86)				41 (65)				1.08 (1.75)		
**Internet usage**					.19				.08				.76	
	<4.00 (n=56)			13.31 (3.67)				32 (57)				1.04 (1.75)		
	≥4.00 (n=59)			14.19 (3.30)				43 (73)				1.14 (1.71)		
**Internet anxiety**					<.001				.19				.17	
	<3.00 (n=58)			15.04 (2.89)				40 (73)				1.32 (2.06)		
	≥3.00 (n=57)			12.58 (3.58)				35 (58)				0.87 (1.39)		
**Physical functioning (Multidimensional Pain Inventory)**					.05				.58				.45	
	<3.90 (n=57)			13.12 (3.40)				36 (63)				1.21 (1.85)		
	≥3.90 (n=58)			14.39 (3.47)				39 (67)				0.97 (1.58)		
**Emotional functioning**														
	**PHQ-8^c^**					.06				.04				.58
		<10.00 (n=55)		13.12 (3.32)				30 (55)				0.99 (1.83)		
		≥10.00 (n=60)		14.35 (3.54)				45 (75)				1.17 (1.63)		
	**GAD-7^d^**					.02				.47				.60
		<8.00 (n=57)		13.03 (3.37)				35 (61)				1.00 (1.55)		
		≥8.00 (n=58)		14.49 (3.50)				40 (69)				1.17 (1.90)		
**Acceptance**					—				<.001				.03	
	<14.00 (n=56)			—				28 (50)				0.72 (1.30)		
	≥14.00 (n=59)			—				47 (80)				1.43 (2.02)		

^a^Low: no school-leaving qualification-higher secondary.

^b^High: highest secondary-university degree.

^c^PHQ-8: Patient Health Questionnaire depression scale.

^d^GAD-7: Generalized Anxiety Disorder Screener 7-item.

## Discussion

### Principal Findings

To the best of our knowledge, this study is the first to examine the impact of AFI on patients’ acceptance, actual uptake, and adherence of an IMI. AFI consisted of a short informational video.

In this study, the average level of acceptance indicated a moderate to high acceptance in the sample (mean 13.76, SD 3.54) with no group differences between IG and CG. This acceptance level is higher than the levels examined in equivalent previous studies [42,46,47]. In these studies, acceptance levels in the intervention group after receiving IMI were at a mean of 11.42 (SD 4.28), 12.17 (SD 4.22), and 10.55 (SD 4.69) in samples of patients with depression [42], pain [47], and diabetes [46], respectively, in routine health care settings. The control groups in these studies displayed substantially lower levels of acceptance below the sum score of 10, indicating a low acceptance level on average. Contrary to previous studies, AFI in our study did not influence acceptance and its predictors, performance expectancy, effort expectancy, social influence, facilitating conditions, or internet anxiety. This might be due to the high baseline level of acceptance in the sample.

The comparatively high acceptance in both groups of this study is potentially due to selective sampling. We recruited the participants from a pool of persons who had already expressed interest in participating in a previous study on ACTonPain. After the end of recruitment for the main study, we invited all persons who were not randomized in the study to participate in this study and to receive ACTonPain as an incentive after completion of the survey. Hence, the participants in this study expressed their interest for ACTonPain twice. Therefore, the level of acceptance most likely reflects the acceptance and uptake in many IMI efficacy studies consisting of a population that is considerably more interested and open to IMIs than the general population [77]. Therefore, our previous work on acceptance in the general population [42,46,47] might give us a more realistic estimate of acceptance. By comparing the acceptance rates throughout the studies, this study quantifies how acceptance and uptake rates can differ between populations in efficacy studies and routine health care settings.

Despite the high level of acceptance, the uptake rate was only moderate at 65.2%. In comparison, the uptake rate in the main evaluation of ACTonPain [22,56] was at 97% in the guided and unguided group, respectively. Furthermore, adherence was considerably low in both groups, again without any influence of AFI. Similar to the results on acceptance, there was no difference between the two groups with regard to uptake and adherence rates, indicating that AFI did not influence uptake rates either. An explanation of why AFI did not influence intervention uptake and adherence is that it targeted acceptance rather than intervention use.

In conclusion, AFI had no effects in a sample with high initial acceptance. This is in line with the assumptions of the Health Action Process Approach (HAPA [78]), which disentangles the processes of intention formation and intention implementation. According to this model, a behavioral intention (ie, acceptance in the UTAUT model) is a necessary but not sufficient precondition of behavior change [79]. In HAPA, three groups of persons are differentiated: nonintenders, intenders, and actors. Each group needs specific behavioral interventions. While nonintenders profit from self-efficacy interventions and information about pros and cons of the behavior change in order to increase behavioral intention, intenders and actors must be provided with concrete help on how to implement their intentions in actual behavior [78]. This includes concrete action planning (when, where, and how to act), coping planning (how to deal with barriers), social support, and action control. By considering these postintentional tasks, HAPA extends the scope of UTAUT and explains the whole range of behavior change, along with the process of intention formation.

Applying the assumptions of HAPA to our sample, an AFI might be the wrong means to increase intervention uptake and adherence. The participants showed high intentions to use an IMI, which means they are classified as intenders in the sense of HAPA. Instead of an AFI, which provides information on efficacy, data security, etc, our participants might have profited more from concrete action and coping planning, action control, and social support. This assumption is supported by a recent study of Zarski et al [80], where planning, out of all the investigated HAPA variables, was the strongest predictor of treatment adherence in highly motivated participants in an IMI.

With most participants only completing the introduction module, adherence is substantially lower in this study than in the evaluation study of the exact same intervention [22,56]. There is little empirical evidence yet on what constitutes an optimal dose of an intervention, for either face-to-face, individual, group, or Web-based interventions. According to the framework of psychological flexibility as the theoretical basis of ACT, the 6 underlying subprocesses are hypothesized to promote higher psychological flexibility as the main goal in ACT [57]. Regarding ACTonPain, this would implicate that participants should have worked on modules 1-6 in order to benefit from ACTonPain. In comparison to this study, participants in the ACTonPain evaluation study completed 5.94 (SD 2.80) and 4.74 (SD 2.89) modules in the guided and unguided groups, respectively. Only 3.5% (4/115) participants completed all 8 modules in this study. In the ACTonPain evaluation study, 60 and 40 participants completed all modules in the guided and unguided groups, respectively. These differences in completion rates are another indicator that participants profit from support in implementing their behavioral intentions. In the ACTonPain evaluation study, participants were enrolled in a broad study procedure, received support from the study team on how to create an account, and were asked to fill out all assessments, including two after randomization. The whole trial procedure might have supported intention implementation via strategies such as reminding prompts or social support [81].

The different findings concerning adherence and dropout not only highlight the influence of guidance and SMS but also the importance of the setting in which participants receive IMIs. Guidance and prompts (eg, through SMS text messages) are two of the most investigated adherence facilitating factors in the research on IMIs, ultimately increasing the effect of the respective IMI [35,82-87]. However, the absence of guidance and SMS Coach alone cannot fully explain the difference in adherence and dropout between the two studies. As there were no following assessments and further administrational contact in this study like in efficacy evaluation trials on IMIs, this study likely imitates a real-life setting. Hence, this finding is consistent with a number of effectiveness trials indicating that the actual use of IMIs is substantially lower when IMIs are implemented in real-life settings [28-31]. Should such findings be replicated in future studies, this could indicate that effect sizes for unguided interventions found in clinical RCTs might be substantially overestimated for what can be expected when embedded in routine health care settings [38]. In conclusion, the findings on adherence and dropout in this study provide an estimate on the use of IMIs when offered in routine health care settings.

In addition to the abovementioned high baseline acceptance, the rather general content of AFI might also explain why AFI was not effective in this study. As discussed in a previous study on acceptance of IMIs in patients with diabetes [66], AFIs tailored to the specific concerns and needs of the respective population might be more effective. The exploratory subgroup analyses in this study showed a trend wherein less anxious (GAD-7<8.00) participants with higher internet anxiety had lower acceptance. Therefore, an AFI with information that is more specific to the characteristics of individuals with lower acceptance might have been more effective. However, as this study was not designed and sufficiently powered to reliably detect heterogeneity in various subgroups, these findings need to be interpreted with caution.

### Limitations

Several limitations in this study are noteworthy. First, the recruiting strategy might have influenced the way the participants filled out the survey, and their answers might have been more socially desirable. Consequently, the results on acceptance and uptake might not be representative for the population of patients with chronic pain, but they are likely to be representative for the population of patients with chronic pain in previous efficacy trials on IMIs for chronic pain. Hence, this study provides information on participants’ acceptance in efficacy studies that can be useful for the interpretation of their respective results. This is especially the case regarding their generalizability to routine clinical practice given that most of these studies are conducted under ideal circumstances with highly specified inclusion and exclusion criteria [77].

In connection with the abovementioned lack of implementation facilitating factors in our AFI, a further limitation of this study is that it is only based on the UTAUT model. The UTAUT model and other equivalent models on the acceptance of IMIs as evaluated in a previous study [75], as well as in some empirical studies [27,53], suggest a relationship between acceptance and IMI use but might not consider sustained use, which is required in IMIs. Therefore, the findings of our study indicate that adherence facilitating factors are crucial even when acceptance is high. Hence, future research is needed to test interventions aimed at increasing adherence. HAPA can serve as an intervention model.

Finally, the reliability of the acceptance scale was relatively low at .71 compared with previous studies (Cronbach alpha ranged from .84 [42] to .87 [46,47]). However, the Cronbach alpha in this study is still in an acceptable range, especially as the scale consists of only 4 items [88].

### Conclusions

Overall, this study yields evidence that patients’ uptake and adherence to an IMI for chronic pain is low, despite high acceptance. The first main contribution of this study is that it shows how acceptance rates can differ between a sample participating in an IMI efficacy trial as represented in this study and a sample collected from a routine health care setting, represented in our previous studies [42,47,66]. This discrepancy should be kept in mind when efficacy trials are interpreted and also when IMIs should be implemented in routine health care settings. In the context of routine health care settings, educational level and motivation are likely to differ from IMI efficacy trial settings [77]. Therefore, effectiveness studies aimed at the clinical target groups in the respective health care settings are needed. As an example, in two studies on an IMI for the treatment [89] and prevention [90] of depression in patients with back pain, recruitment took place following orthopedic rehabilitation. These studies were designed to reach the entire potential target population within a naturalistic setting where the aftercare IMI was implemented. The results of these studies can therefore provide more generalizable results on the effectiveness of IMIs than most of the efficacy trials.

This study also indicates that high acceptance does not guarantee sustainable use of IMIs. Further models, such as the HAPA model, need to be used in order to develop strategies to increase adherence in IMIs. Equivalent to the discussion on acceptance rates, the different settings of efficacy trials and routine health care settings appear to play a crucial role for adherence in IMIs. This might explain the high discrepancy between adherence in this study and the evaluation study of the same intervention [22,56], as well as in a recent meta-analysis on adherence in IMIs for depression [91]. In this review, Van Ballegooijen et al concluded that adherence to guided IMIs (81% of IMI was completed on average) appears to be equal to adherence to face-to-face therapies (84%). Similarly, Christensen et al [92] found dropout rates in IMIs for anxiety and depression to be similar to dropout rates reported in the context of noninternet-based treatments. The findings of this study, however, indicate that when IMIs are offered in routine health care settings, attrition rates might be higher and be a problem specific for IMIs. This is especially the case when guidance as the most important adherence facilitating factor [83] is not provided. Apart from guidance and prompts, it is unclear what specific technological features improve adherence and outcome. Therefore, investigations on attrition and adherence and their underlying mechanisms are needed. In addition to AFIs, engagement facilitating interventions to increase the continuous use of IMIs need to be developed and evaluated. This should comprise constant support systems during the beginning and throughout the use of IMIs, such as continuous monitoring of patients’ health care providers [42]. At this point, it becomes evident that not only IMI users but also their developers and providers need to become involved in order to maximize the acceptance, adherence, and eventually the effectiveness of evidence-based IMIs.

In conclusion, this study shows that acceptance can be much higher in a sample participating in an IMI efficacy trial than in the target population in routine health care settings. Therefore, future research should be conducted within naturalistic settings with more representative samples. Further, strategies to increase adherence in IMIs need to be developed involving IMI users, developers, and providers.

## Abbreviations

AFI: acceptance-facilitating intervention
ACT: Acceptance and Commitment Therapy
ACTonPain: Acceptance and Commitment-based online treatment for chronic pain
CG: control group
DSM-V: Diagnostic and Statistical Manual of Mental Disorders, fifth edition
GAD-7: Generalized Anxiety Disorder Screener 7-item
HAPA: Health Action Process Approach
IG: intervention group
IMI: internet- and mobile-based intervention
PHQ-8: Patient Health Questionnaire depression scale
RCT: randomized controlled trial
SMS: short message service
UTAUT: unified theory of acceptance and use of technology

## Appendix

Multimedia Appendix 1 CONSORT‐EHEALTH checklist (V 1.6.1).
