# Correction: A Web-Based Acceptance-Facilitating Intervention for Identifying Patients’ Acceptance, Uptake, and Adherence of Internet- and Mobile-Based Pain Interventions: Randomized Controlled Trial

**DOI:** 10.2196/12015

**Published:** 2019-02-06

**Authors:** Jiaxi Lin, Bianca Faust, David Daniel Ebert, Lena Krämer, Harald Baumeister

**Affiliations:** 1 Institute of Sports and Sport Science Department of Sport Psychology University of Freiburg Freiburg Germany; 2 Rehabilitation-Center Todtmoos Clinic Wehrawald Todtmoos Germany; 3 Department for Clinical, Neuro- & Developmental Psychology Vrije Universiteit Amsterdam Amsterdam Netherlands; 4 Institute of Psychology Department of Rehabilitation Psychology and Psychotherapy University of Freiburg Freiburg Germany; 5 Institute of Psychology Department of Clinical Psychology and Psychotherapy University of Ulm Ulm Germany

**Keywords:** uptake, acceptance, adherence, eHealth, chronic pain, randomized controlled trial

The authors of “A Web-Based Acceptance-Facilitating Intervention for Identifying Patients’ Acceptance, Uptake, and Adherence of Internet- and Mobile-Based Pain Interventions: Randomized Controlled Trial” (J Med Internet Res 2018;20(8):e244) wish to remove Reference 52 from the sentence "All studies consistently reported low baseline acceptance and an increase in acceptance following AFI [42,46,47,52]" because it does not belong to the group of studies cited in the sentence:

Cranen K, Drossaert CHC, Brinkman ES, Braakman-Jansen ALM, Ijzerman MJ, Vollenbroek-Hutten MMR. An exploration of chronic pain patients' perceptions of home telerehabilitation services. Health Expect 2012 Dec;15(4):339-350. [doi: 10.1111/j.1369-7625.2011.00668.x] [Medline: 21348905]

Reference 52 was removed an all subsequent references were renumbered.

Additionally, the authors have identified a number of references in their paper with display errors, mostly involving names. The corrected references listed below use the new reference number in accordance with the renumbering.

- In Reference 3, author “OPENMinds” has been removed.
- In Reference 20, “van SA” has been changed to “van Straten A”.
- Reference 24 was previously “Paganini S, Lin J, Kahlke F, Buntrock C, Leiding D, Ebert D. A guided and unguided internet- and mobile-based intervention for chronic pahealth economic evaluation alongside a randomized controlled trial. - 3000:- submitted (forthcoming)” and has been changed to “Paganini S, Lin J, Kahlke F, Buntrock C, Leiding D, Ebert DD, et al. A guided and unguided internet- and mobile-based intervention for chronic pain: Health economic evaluation alongside a randomized controlled trial. (forthcoming)”. 
- Reference 37 was previously “Kok R, Beekman A, Cuijpers P, van SA. Adherence to a web-based pre-treatment for phobias in outpatient clinics. Internet Interv. ? 2017;9:45” and has been changed to “Kok R, Beekman A, Cuijpers P, van Straten A. Internet Interventions. 2017 Sep. Adherence to a web-based pre-treatment for phobias in outpatient clinics URL https://www.sciencedirect.com/science/ article/pii/S2214782916300409”.
- In Reference 49, “de NJ” has been changed to “de Nooijer J” and “de VNK” has been changed to “de Vries NK”.
- In Reference 51, “de NJ” has been changed to “de Nooijer J” and “de VNK” has been changed to “de Vries NK”.
- In Reference 55, “Lin M” has been changed to “Lin J” and “Ebert D” has been changed to “Ebert DD”.
- In Reference 67, the author list “Venkatesh, Morris, Davis, Davis” has been changed to “Venkatesh V, Morris MG, Davis GB, Davis FD”.
- In Reference 71, “van DVR” has been changed to “van der Vaart R”.
- In Reference 84, “Van WJJ” has been changed to “Van Wyngaarden JJ”. 
- In Reference 86, “de NJ” has been changed to “de Nooijer J” and “de VNK” has been changed to “de Vries NK”.
- In Reference 90, “van BW” has been changed to “van Ballegooijen W” and “van SA” has been changed to “van Straten A”.

Furthermore, the following items have also been changed:

- In Table 4, the *P* value for “Pain duration” was mistakenly placed in the “<5 years (n=49)” row but has been moved to its intended place in the “Pain duration” row.
- In the “Predictors of Acceptance” subsection within the Methods, “Vance et al” has been changed to “Wilson and Lankton”.
- In the “Statistical Analyses” subsection within the Methods, the sentence “As only those participants who completed the Web-based survey were included in the analysis, there were no missing data in this study” has been deleted.
- In the second paragraph of the “Principal Findings” subsection within the Discussion, the mention of “IMI” in the sentence “In these studies, acceptance levels in the intervention group after receiving IMI were at a mean of…” has been changed to “AFI”.

The correction will appear in the online version of the paper on the JMIR website on February 6, 2019, together with the publication of this correction notice. Because this was made after submission to PubMed, PubMed Central, and other full-text repositories, the corrected article also has been resubmitted to those repositories.

