# Supplementary material for: A Web-Based Acceptance-Facilitating Intervention for Identifying Patients’ Acceptance, Uptake, and Adherence of Internet- and Mobile-Based Pain Interventions: Randomized Controlled Trial
Source: J Med Internet Res. 2018 Aug 21;20(8):e244. doi: 10.2196/jmir.9925 (PMC6123541; doi:10.2196/jmir.9925)
Supplement: Multimedia Appendix 1 [file jmir_v20i8e244_app1.pdf]

CONSORT-EHEALTH (V 1.6.1) - Submission/Publication Form

The CONSORT-EHEALTH checklist is intended for authors of randomized trials evaluating web-based and Internet-based applications/interventions, including mobile interventions, electronic games (incl multiplayer games), social media, certain telehealth applications, and other interactive and/or networked electronic applications. Some of the items (e.g. all subitems under item 5 - description of the intervention) may also be applicable for other study designs.

The goal of the CONSORT EHEALTH checklist and guideline is to be
a) a guide for reporting for authors of RCTs,
b) to form a basis for appraisal of an ehealth trial (in terms of validity)

CONSORT-EHEALTH items/subitems are MANDATORY reporting items for studies published in the Journal of Medical Internet Research and other journals / scientific societies endorsing the checklist.

Items numbered 1., 2., 3., 4a., 4b etc are original CONSORT or CONSORT-NPT (non-pharmacologic treatment) items.
Items with Roman numerals (i., ii, iii, iv etc.) are CONSORT-EHEALTH extensions/clarifications.

As the CONSORT-EHEALTH checklist is still considered in a formative stage, we would ask that you also RATE ON A SCALE OF 1-5 how important/useful you feel each item is FOR THE PURPOSE OF THE CHECKLIST and reporting guideline (optional).

Mandatory reporting items are marked with a red *.
In the textboxes, either copy & paste the relevant sections from your manuscript into this form - please include any quotes from your manuscript in QUOTATION MARKS,
or answer directly by providing additional information not in the manuscript, or elaborating on why the item was not relevant for this study.

YOUR ANSWERS WILL BE PUBLISHED AS A SUPPLEMENTARY FILE TO YOUR PUBLICATION IN JMIR AND ARE CONSIDERED PART OF YOUR PUBLICATION (IF ACCEPTED).
Please fill in these questions diligently. Information will not be copyedited, so please use proper spelling and grammar, use correct capitalization, and avoid abbreviations.

DO NOT FORGET TO SAVE AS PDF _AND_ CLICK THE SUBMIT BUTTON SO YOUR ANSWERS ARE IN OUR DATABASE !!!

Citation Suggestion (if you append the pdf as Appendix we suggest to cite this paper in the caption):
Eysenbach G, CONSORT-EHEALTH Group
CONSORT-EHEALTH: Improving and Standardizing Evaluation Reports of Web-based and Mobile Health Interventions
J Med Internet Res 2011;13(4):e126
URL: http://www.jmir.org/2011/4/e126/
doi: 10.2196/jmir.1923
PMID: 22209829

* Erforderlich

Your name *

First Last

Jiaxi Lin

Primary Affiliation (short), City, Country *

University of Toronto, Toronto, Canada

University of Freiburg, Germany

Your e-mail address *

abc@gmail.com

jiaxi.lin@sport.uni-freiburg.de

Title of your manuscript *
Provide the (draft) title of your manuscript.

A Web-Based Acceptance Facilitating Intervention for Identifying Patients' Acceptance, Uptake, and Adherence of Internet- and Mobile-Based Pain Interventions: A Randomized Controlled Trial

Name of your App/Software/Intervention *
If there is a short and a long/alternate name, write the short name first and add the long name in brackets.

ACTonPain

Evaluated Version (if any)
e.g. "V1", "Release 2017-03-01", "Version 2.0.27913"

Meine Antwort

Language(s) *
What language is the intervention/app in? If multiple languages are available, separate by comma (e.g. "English, French")

German

URL of your Intervention Website or App *
e.g. a direct link to the mobile app on app in appstore (itunes, Google Play), or URL of the website. If the intervention is a DVD or hardware, you can also link to an Amazon page.

https://www.geton-training.de/chronisc

URL of an image/screenshot (optional)

https://www.geton-training.de/chronisc

Accessibility *
Can an enduser access the intervention presently?

- ○ access is free and open
- ● access only for special usergroups, not open
- ○ access is open to everyone, but requires payment/subscription/in-app purchases
- ○ app/intervention no longer accessible
- ○ Sonstiges:

Primary Medical Indication/Disease/Condition *
e.g. "Stress", "Diabetes", or define the target group in brackets after the condition, e.g. "Autism (Parents of children with)", "Alzheimers (Informal Caregivers of)"

chronic pain

Primary Outcomes measured in trial *
comma-separated list of primary outcomes reported in the trial

patients' acceptance, uptake and adhere

Secondary/other outcomes

Are there any other outcomes the intervention is expected to affect?

predictors of acceptance according to UTAUT and internet anxiety

Recommended "Dose" *

What do the instructions for users say on how often the app should be used?

- ○ Approximately Daily
- ◉ Approximately Weekly
- ○ Approximately Monthly
- ○ Approximately Yearly
- ○ "as needed"
- ○ Sonstiges:

Approx. Percentage of Users (starters) still using the app as recommended after 3 months *

- ○ unknown / not evaluated
- ○ 0-10%
- ○ 11-20%
- ○ 21-30%
- ○ 31-40%
- ○ 41-50%
- ○ 51-60%
- ○ 61-70%
- ○ 71%-80%
- ○ 81-90%
- ○ 91-100%
- ◉ Sonstiges: not applicable

Overall, was the app/intervention effective? *

- ◉ yes: all primary outcomes were significantly better in intervention group vs control
- ○ partly: SOME primary outcomes were significantly better in intervention group vs control
- ○ no statistically significant difference between control and intervention
- ○ potentially harmful: control was significantly better than intervention in one or more outcomes
- ○ inconclusive: more research is needed
- ○ Sonstiges:

Article Preparation Status/Stage *

At which stage in your article preparation are you currently (at the time you fill in this form)

- ○ not submitted yet - in early draft status
- ○ not submitted yet - in late draft status, just before submission
- ○ submitted to a journal but not reviewed yet
- ○ submitted to a journal and after receiving initial reviewer comments
- ◉ submitted to a journal and accepted, but not published yet
- ○ published
- ○ Sonstiges:

Journal *

If you already know where you will submit this paper (or if it is already submitted), please provide the journal name (if it is not JMIR, provide the journal name under "other")

- ○ not submitted yet / unclear where I will submit this
- ◉ Journal of Medical Internet Research (JMIR)
- ○ JMIR mHealth and UHealth
- ○ JMIR Serious Games
- ○ JMIR Mental Health
- ○ JMIR Public Health
- ○ JMIR Formative Research
- ○ Other JMIR sister journal
- ○ Sonstiges:

Is this a full powered effectiveness trial or a pilot/feasibility trial? *

- ○ Pilot/feasibility
- ◉ Fully powered

Manuscript tracking number *

If this is a JMIR submission, please provide the manuscript tracking number under "other" (The ms tracking number can be found in the submission acknowledgement email, or when you login as author in JMIR. If the paper is already published in JMIR, then the ms tracking number is the four-digit number at the end of the DOI, to be found at the bottom of each published article in JMIR)

- ○ no ms number (yet) / not (yet) submitted to / published in JMIR
- ◉ Sonstiges: ms#9925

TITLE AND ABSTRACT

1a) TITLE: Identification as a randomized trial in the title

1a) Does your paper address CONSORT item 1a? *

I.e does the title contain the phrase "Randomized Controlled Trial"? (if not, explain the reason under "other")

- ◉ yes
- ○ Sonstiges:

1a-i) Identify the mode of delivery in the title

Identify the mode of delivery. Preferably use “web-based” and/or “mobile” and/or “electronic game” in the title. Avoid ambiguous terms like “online”, “virtual”, “interactive”. Use “Internet-based” only if Intervention includes non-web-based Internet components (e.g. email), use “computer-based” or “electronic” only if offline products are used. Use “virtual” only in the context of “virtual reality” (3-D worlds). Use “online” only in the context of “online support groups”. Complement or substitute product names with broader terms for the class of products (such as “mobile” or “smart phone” instead of “iphone”), especially if the application runs on different platforms.

subitem not at all important ○ ○ ○ ○ ◉ essential

Does your paper address subitem 1a-i? *

Copy and paste relevant sections from manuscript title (include quotes in quotation marks "like this" to indicate direct quotes from your manuscript), or elaborate on this item by providing additional information not in the ms, or briefly explain why the item is not applicable/relevant for your study

A Web-Based Acceptance Facilitating Intervention

1a-ii) Non-web-based components or important co-interventions in title

Mention non-web-based components or important co-interventions in title, if any (e.g., “with telephone support”).

subitem not at all important ○ ○ ○ ○ ◉ essential

Does your paper address subitem 1a-ii?

Copy and paste relevant sections from manuscript title (include quotes in quotation marks "like this" to indicate direct quotes from your manuscript), or elaborate on this item by providing additional information not in the ms, or briefly explain why the item is not applicable/relevant for your study

no

1a-iii) Primary condition or target group in the title

Mention primary condition or target group in the title, if any (e.g., “for children with Type I Diabetes”) Example: A Web-based and Mobile Intervention with Telephone Support for Children with Type I Diabetes: Randomized Controlled Trial

subitem not at all important ○ ○ ○ ○ ◉ essential

Does your paper address subitem 1a-iii? *

Copy and paste relevant sections from manuscript title (include quotes in quotation marks "like this" to indicate direct quotes from your manuscript), or elaborate on this item by providing additional information not in the ms, or briefly explain why the item is not applicable/relevant for your study

"Identifying Patients’ Acceptance, Uptake, and Adherence of Internet- and Mobile-Based Pain Interventions"

1b) ABSTRACT: Structured summary of trial design, methods, results, and conclusions

NPT extension: Description of experimental treatment, comparator, care providers, centers, and blinding status.

1b-i) Key features/functionalities/components of the intervention and comparator in the METHODS section of the ABSTRACT

Mention key features/functionalities/components of the intervention and comparator in the abstract. If possible, also mention theories and principles used for designing the site. Keep in mind the needs of systematic reviewers and indexers by including important synonyms. (Note: Only report in the abstract what the main paper is reporting. If this information is missing from the main body of text, consider adding it)

subitem not at all important ○ ○ ○ ○ ◉ essential

Does your paper address subitem 1b-i? *

Copy and paste relevant sections from the manuscript abstract (include quotes in quotation marks "like this" to indicate direct quotes from your manuscript), or elaborate on this item by providing additional information not in the ms, or briefly explain why the item is not applicable/relevant for your study

"The aim of this study was to identify people's acceptance, uptake, and adherence (primary outcomes) with regard to an IMI for chronic pain and the influence of an information video as an acceptance facilitating intervention (AFI). In this randomized controlled trial with parallel design, we invited 489 individuals with chronic pain to participate in a Web-based survey assessing the acceptance of IMIs with the offer to receive an unguided IMI for chronic pain after completion. Two versions of the Web-based survey (with and without AFI) were randomly sent to two groups: one with AFI (n=245) and one without AFI (n=244). Participants who completed the Web-based survey with or without AFI entered the intervention group (IG) or the control group (CG), respectively."

1b-ii) Level of human involvement in the METHODS section of the ABSTRACT

Clarify the level of human involvement in the abstract, e.g., use phrases like "fully automated" vs. "therapist/nurse/care provider/physician-assisted" (mention number and expertise of providers involved, if any). (Note: Only report in the abstract what the main paper is reporting. If this information is missing from the main body of text, consider adding it)

subitem not at all important ◉ ○ ○ ○ ○ essential

Does your paper address subitem 1b-ii?

Copy and paste relevant sections from the manuscript abstract (include quotes in quotation marks "like this" to indicate direct quotes from your manuscript), or elaborate on this item by providing additional information not in the ms, or briefly explain why the item is not applicable/relevant for your study

no

1b-iii) Open vs. closed, web-based (self-assessment) vs. face-to-face assessments in the METHODS section of the ABSTRACT

Mention how participants were recruited (online vs. offline), e.g., from an open access website or from a clinic or a closed online user group (closed usergroup trial), and clarify if this was a purely web-based trial, or there were face-to-face components (as part of the intervention or for assessment). Clearly say if outcomes were self-assessed through questionnaires (as common in web-based trials). Note: In traditional offline trials, an open trial (open-label trial) is a type of clinical trial in which both the researchers and participants know which treatment is being administered. To avoid confusion, use "blinded" or "unblinded" to indicated the level of blinding instead of "open", as "open" in web-based trials usually refers to "open access" (i.e. participants can self-enrol). (Note: Only report in the abstract what the main paper is reporting. If this information is missing from the main body of text, consider adding it)

subitem not at all important ○ ○ ○ ○ ◉ essential

Does your paper address subitem 1b-iii?

Copy and paste relevant sections from the manuscript abstract (include quotes in quotation marks "like this" to indicate direct quotes from your manuscript), or elaborate on this item by providing additional information not in the ms, or briefly explain why the item is not applicable/relevant for your study

"we invited 489 individuals with chronic pain to participate in a Web-based survey assessing the acceptance of IMIs with the offer to receive an unguided IMI for chronic pain after completion."

1b-iv) RESULTS section in abstract must contain use data

Report number of participants enrolled/assessed in each group, the use/uptake of the intervention (e.g., attrition/adherence metrics, use over time, number of logins etc.), in addition to primary/secondary outcomes. (Note: Only report in the abstract what the main paper is reporting. If this information is missing from the main body of text, consider adding it)

subitem not at all important ○ ○ ○ ○ ◉ essential

Does your paper address subitem 1b-iv?

Copy and paste relevant sections from the manuscript abstract (include quotes in quotation marks "like this" to indicate direct quotes from your manuscript), or elaborate on this item by providing additional information not in the ms, or briefly explain why the item is not applicable/relevant for your study

"Most participants reported moderate (59%, 68/115) to high (31%, 36/115) acceptance, with 10% (11/115) showing low acceptance (IG: mean 13.91, SD 3.47; CG: mean 13.61, SD 3.50). Further, 68% (38/57, IG) and 62% (36/58, CG) had logged into the intervention. In IG and CG, an average of 1.04 (SD 1.51) and 1.14 (SD 1.90) modules were completed, respectively."

1b-v) CONCLUSIONS/DISCUSSION in abstract for negative trials

Conclusions/Discussions in abstract for negative trials: Discuss the primary outcome - if the trial is negative (primary outcome not changed), and the intervention was not used, discuss whether negative results are attributable to lack of uptake and discuss reasons. (Note: Only report in the abstract what the main paper is reporting. If this information is missing from the main body of text, consider adding it)

subitem not at all important ○ ○ ○ ○ ◉ essential

Does your paper address subitem 1b-v?

Copy and paste relevant sections from the manuscript abstract (include quotes in quotation marks "like this" to indicate direct quotes from your manuscript), or elaborate on this item by providing additional information not in the ms, or briefly explain why the item is not applicable/relevant for your study

"The informational video was not effective with regard to acceptance, uptake rate, or adherence. Despite the high acceptance, the uptake rate was only moderate and adherence was remarkably low. This study shows that acceptance can be much higher in a sample participating in an IMI efficacy trial than in the target population in routine health care settings. Thus, future research should focus not only on acceptance and uptake facilitating interventions but also on ways to influence adherence. Further research should be conducted within routine health care settings with more representative samples of the target population."

INTRODUCTION

2a) In INTRODUCTION: Scientific background and explanation of rationale

2a-i) Problem and the type of system/solution

Describe the problem and the type of system/solution that is object of the study: intended as stand-alone intervention vs. incorporated in broader health care program? Intended for a particular patient population? Goals of the intervention, e.g., being more cost-effective to other interventions, replace or complement other solutions? (Note: Details about the intervention are provided in "Methods" under 5)

subitem not at all important ○ ○ ○ ○ ◉ essential

Does your paper address subitem 2a-i? *

Copy and paste relevant sections from the manuscript (include quotes in quotation marks "like this" to indicate direct quotes from your manuscript), or elaborate on this item by providing additional information not in the ms, or briefly explain why the item is not applicable/relevant for your study

"A repeatedly suggested reason for low uptake and adherence is the low level of patients' acceptance of IMIs, conceptualized as the intention to use the intervention [40-42]. Other factors, such as internet usage and anxiety [41,43], uncertainty con-cerning data security, discomfort with use of IMIs and psychological interventions in general, and social influence by friends, family, and health professionals as well as a lack of trust in the effectiveness of IMIs are often reported to influence the acceptance and uptake of IMIs [40,42,44-47]. Aiming at these aspects of acceptance, acceptance facilitating interventions (AFIs) are suggested to reduce patients´ apprehensions and misconceptions about IMIs. They provide trustworthy information on, as well as an introduction to IMIs [40,48-51]."

2a-ii) Scientific background, rationale: What is known about the (type of) system

Scientific background, rationale: What is known about the (type of) system that is the object of the study (be sure to discuss the use of similar systems for other conditions/diagnoses, if appropiate), motivation for the study, i.e. what are the reasons for and what is the context for this specific study, from which stakeholder viewpoint is the study performed, potential impact of findings [2]. Briefly justify the choice of the comparator.

subitem not at all important ○ ○ ○ ○ ◉ essential

Does your paper address subitem 2a-ii? *

Copy and paste relevant sections from the manuscript (include quotes in quotation marks "like this" to indicate direct quotes from your manuscript), or elaborate on this item by providing additional information not in the ms, or briefly explain why the item is not applicable/relevant for your study

"To date, 3 RCTs have investigated the influence of a video-based [42,47] or per-sonal [46] AFI in the clinical population of pain [47], diabetes [46], and primary care patients with depressive symptoms [42]. All studies consistently reported low baseline acceptance and an increase in acceptance following AFI [42,46,47,52]. However, all three studies only examined patients' acceptance and lack more important information on whether AFI effectively increased intervention uptake.
Only two studies have reported on the relationship between IMI acceptance and IMI usage [27,53]. In both studies, a significant association was found between IMI acceptance and usage (log-in and adherence). This finding suggests that AFIs might also influence IMI usage. However, research on the influence of an AFI on interven-tion uptake and adherence is missing."

2b) In INTRODUCTION: Specific objectives or hypotheses

Does your paper address CONSORT subitem 2b? *

Copy and paste relevant sections from the manuscript (include quotes in quotation marks "like this" to indicate direct quotes from your manuscript), or elaborate on this item by providing additional information not in the ms, or briefly explain why the item is not applicable/relevant for your study

"Therefore, in this study we examined whether an informational video (AFI) can increase patients' (1) acceptance of an IMI for chronic pain, (2) uptake of an IMI for chronic pain, and (3) adherence in an IMI for chronic pain.
We expected that AFI would positively increase patients' acceptance as well as the uptake rate and adherence. In addition, we expected that AFI would increase the predictors of acceptance and have a reducing effect on internet anxiety. To examine which factors influence acceptance, uptake rate, and adherence in IMIs, we conducted additional exploratory subgroup analyses."

METHODS

3a) Description of trial design (such as parallel, factorial) including allocation ratio

Does your paper address CONSORT subitem 3a? *

Copy and paste relevant sections from the manuscript (include quotes in quotation marks "like this" to indicate direct quotes from your manuscript), or elaborate on this item by providing additional information not in the ms, or briefly explain why the item is not applicable/relevant for your study

"This was a two-arm pragmatic study using a parallel-group design with balanced (1:1) randomization. The intervention group (IG) received AFI with a subsequent Web-based survey (homepage provided by the University of Freiburg, Germany); the control group (CG) filled out the same Web-based survey without receiving AFI. In this RCT, randomization took place before the assessment of eligibility and inclusion of participants. We chose this procedure as it allowed us to send an invitation email providing a link to the survey in either the IG or CG condition. This is a case of randomization before data are available to confirm the individuals' eligibility without risking bias in the analysis [54]. Therefore, post-randomization exclusions of all non-eligible participants can be regarded as acceptable [54]."

3b) Important changes to methods after trial commencement (such as eligibility criteria), with reasons

Does your paper address CONSORT subitem 3b? *

Copy and paste relevant sections from the manuscript (include quotes in quotation marks "like this" to indicate direct quotes from your manuscript), or elaborate on this item by providing additional information not in the ms, or briefly explain why the item is not applicable/relevant for your study

no

3b-i) Bug fixes, Downtimes, Content Changes

Bug fixes, Downtimes, Content Changes: ehealth systems are often dynamic systems. A description of changes to methods therefore also includes important changes made on the intervention or comparator during the trial (e.g., major bug fixes or changes in the functionality or content) (5-iii) and other "unexpected events" that may have influenced study design such as staff changes, system failures/downtimes, etc. [2].

subitem not at all important ○ ○ ○ ○ ◉ essential

Does your paper address subitem 3b-i?

Copy and paste relevant sections from the manuscript (include quotes in quotation marks "like this" to indicate direct quotes from your manuscript), or elaborate on this item by providing additional information not in the ms, or briefly explain why the item is not applicable/relevant for your study

no

4a) Eligibility criteria for participants

Does your paper address CONSORT subitem 4a? *

Copy and paste relevant sections from the manuscript (include quotes in quotation marks "like this" to indicate direct quotes from your manuscript), or elaborate on this item by providing additional information not in the ms, or briefly explain why the item is not applicable/relevant for your study

"The recruitment took place in September 2015. We sent email invitations to all individuals to participate in this study who had earlier expressed interest in participating in an evaluation study on ACTonPain [22,55]. Individuals in the following categories could not be included in the evaluation study on ACTonPain for the following reasons: (1) screening or baseline assessment not completed or no informed consent for main trial (n=332) or (2) expressed their interest in participating after the target sample size of the main trial was reached (n=157). Applicants for participation in the main trial indicating an elevated risk of suicide were not invited. We assessed the following inclusion criteria based on the Web-based self-report: (1) ≥18 years of age, (2) pain duration≥3 months, (3) sufficient German language skills, and (4) sufficient computer and internet skills to proceed with the Web-based questionnaire. We excluded all participants with an incomplete informed consent form and those not fulfilling the inclusion criteria. "

4a-i) Computer / Internet literacy

Computer / Internet literacy is often an implicit "de facto" eligibility criterion - this should be explicitly clarified.

subitem not at all important ○ ○ ○ ○ ◉ essential

Does your paper address subitem 4a-i?

Copy and paste relevant sections from the manuscript (include quotes in quotation marks "like this" to indicate direct quotes from your manuscript), or elaborate on this item by providing additional information not in the ms, or briefly explain why the item is not applicable/relevant for your study

" (4) sufficient computer and internet skills to proceed with the Web-based questionnaire. "

4a-ii) Open vs. closed, web-based vs. face-to-face assessments:

Open vs. closed, web-based vs. face-to-face assessments: Mention how participants were recruited (online vs. offline), e.g., from an open access website or from a clinic, and clarify if this was a purely web-based trial, or there were face-to-face components (as part of the intervention or for assessment), i.e., to what degree got the study team to know the participant. In online-only trials, clarify if participants were quasi-anonymous and whether having multiple identities was possible or whether technical or logistical measures (e.g., cookies, email confirmation, phone calls) were used to detect/prevent these.

subitem not at all important ○ ○ ○ ○ ◉ essential

Does your paper address subitem 4a-ii? *

Copy and paste relevant sections from the manuscript (include quotes in quotation marks "like this" to indicate direct quotes from your manuscript), or elaborate on this item by providing additional information not in the ms, or briefly explain why the item is not applicable/relevant for your study

"The intervention group (IG) received AFI with a subsequent Web-based survey (homepage provided by the University of Freiburg, Germany); the control group (CG) filled out the same Web-based survey without receiving AFI."

4a-iii) Information giving during recruitment

Information given during recruitment. Specify how participants were briefed for recruitment and in the informed consent procedures (e.g., publish the informed consent documentation as appendix, see also item X26), as this information may have an effect on user self-selection, user expectation and may also bias results.

subitem not at all important ○ ○ ○ ○ ◉ essential

Does your paper address subitem 4a-iii?

Copy and paste relevant sections from the manuscript (include quotes in quotation marks "like this" to indicate direct quotes from your manuscript), or elaborate on this item by providing additional information not in the ms, or briefly explain why the item is not applicable/relevant for your study

"Reading and providing online informed consent and answering the survey took about 20-30 minutes. "

4b) Settings and locations where the data were collected

Does your paper address CONSORT subitem 4b? *

Copy and paste relevant sections from the manuscript (include quotes in quotation marks "like this" to indicate direct quotes from your manuscript), or elaborate on this item by providing additional information not in the ms, or briefly explain why the item is not applicable/relevant for your study

"We sent email invitations to all individuals to participate in this study who had earlier expressed interest in participating in an evaluation study on ACTonPain [22,55]. Individuals in the following categories could not be included in the evaluation study on ACTonPain for the following reasons: (1) screening or baseline assessment not completed or no informed consent for main trial (n=332) or (2) expressed their interest in participating after the target sample size of the main trial was reached (n=157). Applicants for participation in the main trial indicating an elevated risk of suicide were not invited."

4b-i) Report if outcomes were (self-)assessed through online questionnaires

Clearly report if outcomes were (self-)assessed through online questionnaires (as common in web-based trials) or otherwise.

subitem not at all important ○ ○ ○ ○ ◉ essential

Does your paper address subitem 4b-i? *

Copy and paste relevant sections from the manuscript (include quotes in quotation marks "like this" to indicate direct quotes from your manuscript), or elaborate on this item by providing additional information not in the ms, or briefly explain why the item is not applicable/relevant for your study

"Reading and providing online informed consent and answering the survey took about 20-30 minutes. "

4b-ii) Report how institutional affiliations are displayed

Report how institutional affiliations are displayed to potential participants [on ehealth media], as affiliations with prestigious hospitals or universities may affect volunteer rates, use, and reactions with regards to an intervention.(Not a required item – describe only if this may bias results)

subitem not at all important ○ ○ ○ ○ ◉ essential

Does your paper address subitem 4b-ii?

Copy and paste relevant sections from the manuscript (include quotes in quotation marks "like this" to indicate direct quotes from your manuscript), or elaborate on this item by providing additional information not in the ms, or briefly explain why the item is not applicable/relevant for your study

"The intervention group (IG) received AFI with a subsequent Web-based survey (homepage provided by the University of Freiburg, Germany); the control group (CG) filled out the same Web-based survey without receiving AFI. "

5) The interventions for each group with sufficient details to allow replication, including how and when they were actually administered

5-i) Mention names, credential, affiliations of the developers, sponsors, and owners

Mention names, credential, affiliations of the developers, sponsors, and owners [6] (if authors/evaluators are owners or developer of the software, this needs to be declared in a "Conflict of interest" section or mentioned elsewhere in the manuscript).

subitem not at all important	○	○	○	○	◉	essential

Does your paper address subitem 5-i?

Copy and paste relevant sections from the manuscript (include quotes in quotation marks "like this" to indicate direct quotes from your manuscript), or elaborate on this item by providing additional information not in the ms, or briefly explain why the item is not applicable/relevant for your study

"The intervention group (IG) received AFI with a subsequent Web-based survey (homepage provided by the University of Freiburg, Germany); the control group (CG) filled out the same Web-based survey without receiving AFI. "

5-ii) Describe the history/development process

Describe the history/development process of the application and previous formative evaluations (e.g., focus groups, usability testing), as these will have an impact on adoption/use rates and help with interpreting results.

subitem not at all important	○	○	○	○	◉	essential

Does your paper address subitem 5-ii?

Copy and paste relevant sections from the manuscript (include quotes in quotation marks "like this" to indicate direct quotes from your manuscript), or elaborate on this item by providing additional information not in the ms, or briefly explain why the item is not applicable/relevant for your study

"AFI consisted of a 3-minute introductory and information video to ACTonPain with screenshots of the program in order to improve patients' acceptance. Figure 2 pro-vides screenshots of AFI. We designed the content of the intervention to address the aforementioned barriers and drivers of acceptance. We conceptualized the video based on our previous AFIs that showed to be effective in increasing acceptance [42,47,65]. Our AFI is an adopted version of AFI used in a former study with individuals with chronic pain [47] with a specific introduction to ACTonPain. The content of the video comprised information on (1) the effectiveness of IMIs, (2) data security and anonymity in IMIs, (3) various advantages of IMIs (eg, ease and comfort of use, flexible time management), (4) the possibility of receiving technical support, and (5) assistance during the program. Furthermore, the video presented the process for using ACTonPain, encompassing the log-in or log-off processes and an overview of the modules and different features (audio files, video clips, and homework assignments)."

5-iii) Revisions and updating

Revisions and updating. Clearly mention the date and/or version number of the application/intervention (and comparator, if applicable) evaluated, or describe whether the intervention underwent major changes during the evaluation process, or whether the development and/or content was “frozen” during the trial. Describe dynamic components such as news feeds or changing content which may have an impact on the replicability of the intervention (for unexpected events see item 3b).

subitem not at all important ○ ○ ○ ○ ◉ essential

Does your paper address subitem 5-iii?

Copy and paste relevant sections from the manuscript (include quotes in quotation marks "like this" to indicate direct quotes from your manuscript), or elaborate on this item by providing additional information not in the ms, or briefly explain why the item is not applicable/relevant for your study

"Our AFI is an adopted version of AFI used in a former study with individuals with chronic pain [47] with a specific introduction to ACTonPain. The content of the video comprised information on (1) the effectiveness of IMIs, (2) data security and anonymity in IMIs, (3) various advantages of IMIs (eg, ease and comfort of use, flexible time management), (4) the possibility of receiving technical support, and (5) assistance during the program. Furthermore, the video presented the process for using ACTonPain, encompassing the log-in or log-off processes and an overview of the modules and different features (audio files, video clips, and homework assignments)."

5-iv) Quality assurance methods

Provide information on quality assurance methods to ensure accuracy and quality of information provided [1], if applicable.

subitem not at all important ○ ○ ◉ ○ ○ essential

Does your paper address subitem 5-iv?

Copy and paste relevant sections from the manuscript (include quotes in quotation marks "like this" to indicate direct quotes from your manuscript), or elaborate on this item by providing additional information not in the ms, or briefly explain why the item is not applicable/relevant for your study

Meine Antwort

5-v) Ensure replicability by publishing the source code, and/or providing screenshots/screen-capture video, and/or providing flowcharts of the algorithms used

Ensure replicability by publishing the source code, and/or providing screenshots/screen-capture video, and/or providing flowcharts of the algorithms used. Replicability (i.e., other researchers should in principle be able to replicate the study) is a hallmark of scientific reporting.

subitem not at all important ○ ○ ○ ○ ◉ essential

Does your paper address subitem 5-v?

Copy and paste relevant sections from the manuscript (include quotes in quotation marks "like this" to indicate direct quotes from your manuscript), or elaborate on this item by providing additional information not in the ms, or briefly explain why the item is not applicable/relevant for your study

"Figure 1. Flow chart. IG: intervention group; CG: control group."
"Figure 2. Screenshots of the acceptance facilitating intervention. Screenshot 1: content of an online pain intervention. Screenshot 2: introduction to Acceptance and Commitment-based online treatment for chronic pain (ACTonPain) log-in page. Screenshot 3: intro-duction to ACTonPain features. Screenshot 4: information concerning data security."

5-vi) Digital preservation

Digital preservation: Provide the URL of the application, but as the intervention is likely to change or disappear over the course of the years; also make sure the intervention is archived (Internet Archive, webcitation.org, and/or publishing the source code or screenshots/videos alongside the article). As pages behind login screens cannot be archived, consider creating demo pages which are accessible without login.

subitem not at all important ○ ○ ○ ○ ◉ essential

Does your paper address subitem 5-vi?

Copy and paste relevant sections from the manuscript (include quotes in quotation marks "like this" to indicate direct quotes from your manuscript), or elaborate on this item by providing additional information not in the ms, or briefly explain why the item is not applicable/relevant for your study

Meine Antwort

5-vii) Access

Access: Describe how participants accessed the application, in what setting/context, if they had to pay (or were paid) or not, whether they had to be a member of specific group. If known, describe how participants obtained "access to the platform and Internet" [1]. To ensure access for editors/reviewers/readers, consider to provide a "backdoor" login account or demo mode for reviewers/readers to explore the application (also important for archiving purposes, see vi).

subitem not at all important ○ ○ ○ ○ ◉ essential

Does your paper address subitem 5-vii? *

Copy and paste relevant sections from the manuscript (include quotes in quotation marks "like this" to indicate direct quotes from your manuscript), or elaborate on this item by providing additional information not in the ms, or briefly explain why the item is not applicable/relevant for your study

"Reading and providing online informed consent and answering the survey took about 20-30 minutes. After completing the survey, the participants could choose to receive the unguided version of ACTonPain [22,55] by providing their email ad-dress in order to create to access ACTonPain."

5-viii) Mode of delivery, features/functionalities/components of the intervention and comparator, and the theoretical framework

Describe mode of delivery, features/functionalities/components of the intervention and comparator, and the theoretical framework [6] used to design them (instructional strategy [1], behaviour change techniques, persuasive features, etc., see e.g., [7, 8] for terminology). This includes an in-depth description of the content (including where it is coming from and who developed it) [1]," whether [and how] it is tailored to individual circumstances and allows users to track their progress and receive feedback" [6]. This also includes a description of communication delivery channels and – if computer-mediated communication is a component – whether communication was synchronous or asynchronous [6]. It also includes information on presentation strategies [1], including page design principles, average amount of text on pages, presence of hyperlinks to other resources, etc. [1].

subitem not at all important ○ ○ ○ ○ ◉ essential

Does your paper address subitem 5-viii? *

Copy and paste relevant sections from the manuscript (include quotes in quotation marks "like this" to indicate direct quotes from your manuscript), or elaborate on this item by providing additional information not in the ms, or briefly explain why the item is not applicable/relevant for your study

"The intervention group (IG) received AFI with a subsequent Web-based survey (homepage provided by the University of Freiburg, Germany); the control group (CG) filled out the same Web-based survey without receiving AFI. In this RCT, ran-domization took place before the assessment of eligibility and inclusion of partici-pants. We chose this procedure as it allowed us to send an invitation email provid-ing a link to the survey in either the IG or CG condition. "

5-ix) Describe use parameters

Describe use parameters (e.g., intended "doses" and optimal timing for use). Clarify what instructions or recommendations were given to the user, e.g., regarding timing, frequency, heaviness of use, if any, or was the intervention used ad libitum.

subitem not at all important ○ ○ ○ ○ ◉ essential

Does your paper address subitem 5-ix?

Copy and paste relevant sections from the manuscript (include quotes in quotation marks "like this" to indicate direct quotes from your manuscript), or elaborate on this item by providing additional information not in the ms, or briefly explain why the item is not applicable/relevant for your study

"ACTonPain consists of an introduction and 7 consecutive modules. The intervention targets core change processes proposed by Hayes et al [56] and is described in more detail by Lin et al [55]. Participants were advised to complete one session per week with a completion time of approximately 60 minutes. "

5-x) Clarify the level of human involvement

Clarify the level of human involvement (care providers or health professionals, also technical assistance) in the e-intervention or as co-intervention (detail number and expertise of professionals involved, if any, as well as "type of assistance offered, the timing and frequency of the support, how it is initiated, and the medium by which the assistance is delivered". It may be necessary to distinguish between the level of human involvement required for the trial, and the level of human involvement required for a routine application outside of a RCT setting (discuss under item 21 – generalizability).

subitem not at all important ○ ○ ○ ○ ◉ essential

Does your paper address subitem 5-x?

Copy and paste relevant sections from the manuscript (include quotes in quotation marks "like this" to indicate direct quotes from your manuscript), or elaborate on this item by providing additional information not in the ms, or briefly explain why the item is not applicable/relevant for your study

"After completing the questionnaire, participants were invited to receive ACTonPain treatment in an unguided version and without short message service (SMS) text messages (SMS Coach). This version of ACTonPain was provided without any human support and should be therefore of special interest for public health services due to its high scalability and low costs. "

5-xi) Report any prompts/reminders used

Report any prompts/reminders used: Clarify if there were prompts (letters, emails, phone calls, SMS) to use the application, what triggered them, frequency etc. It may be necessary to distinguish between the level of prompts/reminders required for the trial, and the level of prompts/reminders for a routine application outside of a RCT setting (discuss under item 21 – generalizability).

subitem not at all important ○ ○ ○ ○ ◉ essential

Does your paper address subitem 5-xi? *

Copy and paste relevant sections from the manuscript (include quotes in quotation marks "like this" to indicate direct quotes from your manuscript), or elaborate on this item by providing additional information not in the ms, or briefly explain why the item is not applicable/relevant for your study

"After completing the questionnaire, participants were invited to receive ACTonPain treatment in an unguided version and without short message service (SMS) text messages (SMS Coach). "

5-xii) Describe any co-interventions (incl. training/support)

Describe any co-interventions (incl. training/support): Clearly state any interventions that are provided in addition to the targeted eHealth intervention, as ehealth intervention may not be designed as stand-alone intervention. This includes training sessions and support [1]. It may be necessary to distinguish between the level of training required for the trial, and the level of training for a routine application outside of a RCT setting (discuss under item 21 – generalizability.

subitem not at all important ○ ○ ○ ○ ◉ essential

Does your paper address subitem 5-xii? *

Copy and paste relevant sections from the manuscript (include quotes in quotation marks "like this" to indicate direct quotes from your manuscript), or elaborate on this item by providing additional information not in the ms, or briefly explain why the item is not applicable/relevant for your study

"All participants had full access to treatment as usual."

6a) Completely defined pre-specified primary and secondary outcome measures, including how and when they were assessed

Does your paper address CONSORT subitem 6a? *

Copy and paste relevant sections from the manuscript (include quotes in quotation marks "like this" to indicate direct quotes from your manuscript), or elaborate on this item by providing additional information not in the ms, or briefly explain why the item is not applicable/relevant for your study

"Primary Outcomes
The primary outcomes were acceptance, uptake, and adherence.
Acceptance: We operationalized acceptance on the basis of the well-established uni-fied theory of acceptance and use of technology (UTAUT [66,67]). This framework provides a reliable theoretical basis of drivers and barriers for users' acceptance of information technology [66-68] and has been used in numerous IMIs studies [27,47,65,69-72]. The UTAUT model postulates acceptance as the intention to use technology and the proximal predictor for actual use [73].
The items of the UTAUT acceptance were developed based on previous studies [46,47]. The sum score of the scale ranges from 4 to 20, and the 3 levels of ac-ceptance can be categorized: low (sum score: 4-9), moderate (sum score: 10-15), and high (sum score: 16-20). The Cronbach alpha in this study was relatively low at.71. Table 1 provides an overview of the items for acceptance and predictors of acceptance (see secondary outcomes) in this study, including their scales.
Uptake: We operationalized uptake as log-in (yes or no) to IMI assessed 4 months after intervention access. The period of 4 months was chosen, as this should have been enough time for the participants to start with the intervention and work through all 8 modules. We assumed that 4 months after intervention access is a reasonable time to assess uptake and adherence.
Adherence: We operationalized adherence as the number of completed modules of the intervention assessed 4 months after intervention access.

Table 1. Items of acceptance and predictors of acceptance according to the unified theory of acceptance and use of technology model.

Secondary Outcomes
The secondary outcomes were the predictors of acceptance according to UTAUT as well as internet anxiety.
Predictors of acceptance: According to the UTAUT model, there are 4 key predictors of either the behavioral intention or usage behavior of IT: performance expec-tancy, effort expectancy, social influence, and facilitating conditions [67]. The items measuring the construct's performance expectancy and effort expectancy were drawn from Vance et al [74]. The items for social influence and facilitating conditions were adapted from Venkatesh et al [67].
Internet anxiety: Two items for internet anxiety were adapted from Venkatesh et al [67] ("1. The internet is something threatening to me" and "2. I am afraid of making an irrevocable mistake while using the internet"). The items were rated on a 5-point Likert scale ranging from 1 "does not apply at all" to 5 "applies completely." The Cronbach alpha in this study was at.69."

6a-i) Online questionnaires: describe if they were validated for online use and apply CHERRIES items to describe how the questionnaires were designed/deployed

If outcomes were obtained through online questionnaires, describe if they were validated for online use and apply CHERRIES items to describe how the questionnaires were designed/deployed [9].

subitem not at all important ○ ○ ○ ○ ◉ essential

Does your paper address subitem 6a-i?

Copy and paste relevant sections from manuscript text

see above

6a-ii) Describe whether and how "use" (including intensity of use/dosage) was defined/measured/monitored

Describe whether and how "use" (including intensity of use/dosage) was defined/measured/monitored (logins, logfile analysis, etc.). Use/adoption metrics are important process outcomes that should be reported in any ehealth trial.

subitem not at all important ○ ○ ○ ○ ◉ essential

Does your paper address subitem 6a-ii?

Copy and paste relevant sections from manuscript text

see above

6a-iii) Describe whether, how, and when qualitative feedback from participants was obtained

Describe whether, how, and when qualitative feedback from participants was obtained (e.g., through emails, feedback forms, interviews, focus groups).

subitem not at all important ○ ○ ○ ○ ○ essential

Does your paper address subitem 6a-iii?

Copy and paste relevant sections from manuscript text

Meine Antwort

6b) Any changes to trial outcomes after the trial commenced, with reasons

Does your paper address CONSORT subitem 6b? *

Copy and paste relevant sections from the manuscript (include quotes in quotation marks "like this" to indicate direct quotes from your manuscript), or elaborate on this item by providing additional information not in the ms, or briefly explain why the item is not applicable/relevant for your study

no

7a) How sample size was determined

NPT: When applicable, details of whether and how the clustering by care provides or centers was addressed

7a-i) Describe whether and how expected attrition was taken into account when calculating the sample size

Describe whether and how expected attrition was taken into account when calculating the sample size.

subitem not at all important ○ ○ ○ ○ ◉ essential

Does your paper address subitem 7a-i?

Copy and paste relevant sections from manuscript title (include quotes in quotation marks "like this" to indicate direct quotes from your manuscript), or elaborate on this item by providing additional information not in the ms, or briefly explain why the item is not applicable/relevant for your study

"To detect differences between IG and CG regarding acceptance, uptake, adherence, and the predictors of acceptance as well as internet anxiety, we conducted t-tests for independent samples and chi-square tests. In case of significant group differences, standardized mean differences (Cohen d) with a 95% CI were computed to quantify the effect. As this study includes multiple primary outcomes, we used a Bonferroni adjustment for the p-values of.016 (3 tests at an alpha level of.05). This procedure resulted in sufficient statistical power with the sample to detect differences between the two conditions that were larger than Cohen d=0.65."

7b) When applicable, explanation of any interim analyses and stopping guidelines

Does your paper address CONSORT subitem 7b? *

Copy and paste relevant sections from the manuscript (include quotes in quotation marks "like this" to indicate direct quotes from your manuscript), or elaborate on this item by providing additional information not in the ms, or briefly explain why the item is not applicable/relevant for your study

not applicable

8a) Method used to generate the random allocation sequence

NPT: When applicable, how care providers were allocated to each trial group

Does your paper address CONSORT subitem 8a? *

Copy and paste relevant sections from the manuscript (include quotes in quotation marks "like this" to indicate direct quotes from your manuscript), or elaborate on this item by providing additional information not in the ms, or briefly explain why the item is not applicable/relevant for your study

"For allocation to IG or CG, a computer-generated list of random numbers with randomly varying block sizes of 4, 6, and 8 was used by BF (www.sealedenvelope.com). IG watched an AFI video before answering the Web-based questionnaire. CG filled out the questionnaire immediately. Out of 489 potential participants, 115 provided informed consent and fulfilled the inclusion criteria (Figure 1)."

8b) Type of randomisation; details of any restriction (such as blocking and block size)

Does your paper address CONSORT subitem 8b? *

Copy and paste relevant sections from the manuscript (include quotes in quotation marks "like this" to indicate direct quotes from your manuscript), or elaborate on this item by providing additional information not in the ms, or briefly explain why the item is not applicable/relevant for your study

"For allocation to IG or CG, a computer-generated list of random numbers with randomly varying block sizes of 4, 6, and 8 was used by BF (www.sealedenvelope.com)."

9) Mechanism used to implement the random allocation sequence (such as sequentially numbered containers), describing any steps taken to conceal the sequence until interventions were assigned

Does your paper address CONSORT subitem 9? *

Copy and paste relevant sections from the manuscript (include quotes in quotation marks "like this" to indicate direct quotes from your manuscript), or elaborate on this item by providing additional information not in the ms, or briefly explain why the item is not applicable/relevant for your study

"The intervention group (IG) received AFI with a subsequent Web-based survey (homepage provided by the University of Freiburg, Germany); the control group (CG) filled out the same Web-based survey without receiving AFI. In this RCT, randomization took place before the assessment of eligibility and inclusion of partici-pants. We chose this procedure as it allowed us to send an invitation email provid-ing a link to the survey in either the IG or CG condition. This is a case of randomiza-tion before data are available to confirm the individuals' eligibility without risking bias in the analysis [54]."
"For allocation to IG or CG, a computer-generated list of random numbers with randomly varying block sizes of 4, 6, and 8 was used by BF (www.sealedenvelope.com)."

10) Who generated the random allocation sequence, who enrolled participants, and who assigned participants to interventions

Does your paper address CONSORT subitem 10? *

Copy and paste relevant sections from the manuscript (include quotes in quotation marks "like this" to indicate direct quotes from your manuscript), or elaborate on this item by providing additional information not in the ms, or briefly explain why the item is not applicable/relevant for your study

"For allocation to IG or CG, a computer-generated list of random numbers with randomly varying block sizes of 4, 6, and 8 was used by BF (www.sealedenvelope.com)."

11a) If done, who was blinded after assignment to interventions (for example, participants, care providers, those assessing outcomes) and how

NPT: Whether or not administering co-interventions were blinded to group assignment

11a-i) Specify who was blinded, and who wasn't

Specify who was blinded, and who wasn't. Usually, in web-based trials it is not possible to blind the participants [1, 3] (this should be clearly acknowledged), but it may be possible to blind outcome assessors, those doing data analysis or those administering co-interventions (if any).

subitem not at all important ○ ○ ○ ○ ◉ essential

Does your paper address subitem 11a-i? *

Copy and paste relevant sections from the manuscript (include quotes in quotation marks "like this" to indicate direct quotes from your manuscript), or elaborate on this item by providing additional information not in the ms, or briefly explain why the item is not applicable/relevant for your study

Participants could not be blinded to study conditions, the allocation was concealed from participants and researchers involved in recruitment

11a-ii) Discuss e.g., whether participants knew which intervention was the "intervention of interest" and which one was the "comparator"

Informed consent procedures (4a-ii) can create biases and certain expectations - discuss e.g., whether participants knew which intervention was the "intervention of interest" and which one was the "comparator".

subitem not at all important ○ ○ ○ ○ ◉ essential

Does your paper address subitem 11a-ii?

Copy and paste relevant sections from the manuscript (include quotes in quotation marks "like this" to indicate direct quotes from your manuscript), or elaborate on this item by providing additional information not in the ms, or briefly explain why the item is not applicable/relevant for your study

participants were blinded and did not know whether they were receiving AFI or not

11b) If relevant, description of the similarity of interventions

(this item is usually not relevant for ehealth trials as it refers to similarity of a placebo or sham intervention to a active medication/intervention)

Does your paper address CONSORT subitem 11b? *

Copy and paste relevant sections from the manuscript (include quotes in quotation marks "like this" to indicate direct quotes from your manuscript), or elaborate on this item by providing additional information not in the ms, or briefly explain why the item is not applicable/relevant for your study

not applicable

12a) Statistical methods used to compare groups for primary and secondary outcomes

NPT: When applicable, details of whether and how the clustering by care providers or centers was addressed

Does your paper address CONSORT subitem 12a? *

Copy and paste relevant sections from the manuscript (include quotes in quotation marks "like this" to indicate direct quotes from your manuscript), or elaborate on this item by providing additional information not in the ms, or briefly explain why the item is not applicable/relevant for your study

"To detect differences between IG and CG regarding acceptance, uptake, adherence, and the predictors of acceptance as well as internet anxiety, we conducted t-tests for independent samples and chi-square tests. In case of significant group differences, standardized mean differences (Cohen d) with a 95% CI were computed to quantify the effect. As this study includes multiple primary outcomes, we used a Bonferroni adjustment for the p-values of.016 (3 tests at an alpha level of.05). This procedure resulted in sufficient statistical power with the sample to detect differences between the two conditions that were larger than Cohen d=0.65."

12a-i) Imputation techniques to deal with attrition / missing values

Imputation techniques to deal with attrition / missing values: Not all participants will use the intervention/comparator as intended and attrition is typically high in ehealth trials. Specify how participants who did not use the application or dropped out from the trial were treated in the statistical analysis (a complete case analysis is strongly discouraged, and simple imputation techniques such as LOCF may also be problematic [4]).

subitem not at all important ○ ○ ○ ○ ◉ essential

Does your paper address subitem 12a-i? *

Copy and paste relevant sections from the manuscript (include quotes in quotation marks "like this" to indicate direct quotes from your manuscript), or elaborate on this item by providing additional information not in the ms, or briefly explain why the item is not applicable/relevant for your study

"The descriptive statistics were based on nonimputed data, while all following analyses were conducted after multiple imputations with 20 imputations using the imputation algorithm implemented in SPSS (intention-to-treat analysis)."

12b) Methods for additional analyses, such as subgroup analyses and adjusted analyses

Does your paper address CONSORT subitem 12b? *

Copy and paste relevant sections from the manuscript (include quotes in quotation marks "like this" to indicate direct quotes from your manuscript), or elaborate on this item by providing additional information not in the ms, or briefly explain why the item is not applicable/relevant for your study

"To examine potential subgroup differences (age, gender, education, pain duration and intensity, prior or present psychological intervention, internet usage and anxiety, and physical and emotional functioning) regarding acceptance, uptake, and adherence, exploratory analyses are provided (mean, SD, t-tests, and chi-square test). For this purpose, variables were dichotomized using defined cutoffs (gender, pain duration, education, and psychological intervention) or a median split (age, pain intensity, internet usage and anxiety, physical and emotional functioning, and level of acceptance regarding uptake and adherence). Note that the results of the subgroup analyses and analysis on secondary outcomes are exploratory and underpowered; adjusting for multiple testing would not be meaningful [75]."

X26) REB/IRB Approval and Ethical Considerations [recommended as subheading under "Methods"] (not a CONSORT item)

X26-i) Comment on ethics committee approval

subitem not at all important ○ ○ ○ ○ ◉ essential

Does your paper address subitem X26-i?

Copy and paste relevant sections from the manuscript (include quotes in quotation marks "like this" to indicate direct quotes from your manuscript), or elaborate on this item by providing additional information not in the ms, or briefly explain why the item is not applicable/relevant for your study

"This study is linked to an outcome evaluation study with the German Clinical Trial Registration (DRKS): DRKS00006183 and approved by the ethics committee of the Albert-Ludwigs-University of Freiburg. "

x26-ii) Outline informed consent procedures

Outline informed consent procedures e.g., if consent was obtained offline or online (how? Checkbox, etc.?), and what information was provided (see 4a-ii). See [6] for some items to be included in informed consent documents.

subitem not at all important ○ ○ ○ ○ ◉ essential

Does your paper address subitem X26-ii?

Copy and paste relevant sections from the manuscript (include quotes in quotation marks "like this" to indicate direct quotes from your manuscript), or elaborate on this item by providing additional information not in the ms, or briefly explain why the item is not applicable/relevant for your study

"Reading and providing online informed consent and answering the survey took about 20-30 minutes. "

X26-iii) Safety and security procedures

Safety and security procedures, incl. privacy considerations, and any steps taken to reduce the likelihood or detection of harm (e.g., education and training, availability of a hotline)

subitem not at all important ○ ○ ○ ○ ○ essential

Does your paper address subitem X26-iii?

Copy and paste relevant sections from the manuscript (include quotes in quotation marks "like this" to indicate direct quotes from your manuscript), or elaborate on this item by providing additional information not in the ms, or briefly explain why the item is not applicable/relevant for your study

Meine Antwort

RESULTS

13a) For each group, the numbers of participants who were randomly assigned, received intended treatment, and were analysed for the primary outcome

NPT: The number of care providers or centers performing the intervention in each group and the number of patients treated by each care provider in each center

Does your paper address CONSORT subitem 13a? *

Copy and paste relevant sections from the manuscript (include quotes in quotation marks "like this" to indicate direct quotes from your manuscript), or elaborate on this item by providing additional information not in the ms, or briefly explain why the item is not applicable/relevant for your study

see Figure 1. Flow chart. IG: intervention group; CG: control group.
"Of 489 persons, 141 responded to the invitation. After we excluded those who did not provide informed consent (n=22) or did not fulfill the inclusion criteria (n=4), we included 57 and 58 participants in IG and CG, respectively. The missing value was between 0% and 5.7% per variable, and Little's Missing Completely at Random test indicated that the data were missing at random (χ241=45.31, P=.30)."

13b) For each group, losses and exclusions after randomisation, together with reasons

Does your paper address CONSORT subitem 13b? (NOTE: Preferably, this is shown in a CONSORT flow diagram) *

Copy and paste relevant sections from the manuscript (include quotes in quotation marks "like this" to indicate direct quotes from your manuscript), or elaborate on this item by providing additional information not in the ms, or briefly explain why the item is not applicable/relevant for your study

see Figure 1. Flow chart. IG: intervention group; CG: control group.

13b-i) Attrition diagram

Strongly recommended: An attrition diagram (e.g., proportion of participants still logging in or using the intervention/comparator in each group plotted over time, similar to a survival curve) or other figures or tables demonstrating usage/dose/engagement.

subitem not at all important ○ ○ ○ ○ ◉ essential

Does your paper address subitem 13b-i?

Copy and paste relevant sections from the manuscript or cite the figure number if applicable (include quotes in quotation marks "like this" to indicate direct quotes from your manuscript), or elaborate on this item by providing additional information not in the ms, or briefly explain why the item is not applicable/relevant for your study

see Figure 1. Flow chart. IG: intervention group; CG: control group.

14a) Dates defining the periods of recruitment and follow-up

Does your paper address CONSORT subitem 14a? *

Copy and paste relevant sections from the manuscript (include quotes in quotation marks "like this" to indicate direct quotes from your manuscript), or elaborate on this item by providing additional information not in the ms, or briefly explain why the item is not applicable/relevant for your study

"The recruitment took place in September 2015"
"Uptake: We operationalized uptake as login (yes or no) to the IMI assessed 4 months after intervention access.
Adherence: We operationalized adherence as number of completed modules of the intervention assessed 4 months after intervention access. "

14a-i) Indicate if critical “secular events” fell into the study period

Indicate if critical “secular events” fell into the study period, e.g., significant changes in Internet resources available or “changes in computer hardware or Internet delivery resources”

subitem not at all important ○ ○ ○ ○ ◉ essential

Does your paper address subitem 14a-i?

Copy and paste relevant sections from the manuscript (include quotes in quotation marks "like this" to indicate direct quotes from your manuscript), or elaborate on this item by providing additional information not in the ms, or briefly explain why the item is not applicable/relevant for your study

There were no “secular events” dring the study period

14b) Why the trial ended or was stopped (early)

Does your paper address CONSORT subitem 14b? *

Copy and paste relevant sections from the manuscript (include quotes in quotation marks "like this" to indicate direct quotes from your manuscript), or elaborate on this item by providing additional information not in the ms, or briefly explain why the item is not applicable/relevant for your study

The trial stopped - as planned - when the invitation mail was sent to all potential participants

15) A table showing baseline demographic and clinical characteristics for each group

NPT: When applicable, a description of care providers (case volume, qualification, expertise, etc.) and centers (volume) in each group

Does your paper address CONSORT subitem 15? *

Copy and paste relevant sections from the manuscript (include quotes in quotation marks "like this" to indicate direct quotes from your manuscript), or elaborate on this item by providing additional information not in the ms, or briefly explain why the item is not applicable/relevant for your study

see Table 2. Sociodemographic and clinical characteristics and internet usage.

15-i) Report demographics associated with digital divide issues

In ehealth trials it is particularly important to report demographics associated with digital divide issues, such as age, education, gender, social-economic status, computer/Internet/ehealth literacy of the participants, if known.

subitem not at all important ○ ○ ○ ○ ◉ essential

Does your paper address subitem 15-i? *

Copy and paste relevant sections from the manuscript (include quotes in quotation marks "like this" to indicate direct quotes from your manuscript), or elaborate on this item by providing additional information not in the ms, or briefly explain why the item is not applicable/relevant for your study

Table 2. Sociodemographic and clinical characteristics and internet usage.

16) For each group, number of participants (denominator) included in each analysis and whether the analysis was by original assigned groups

16-i) Report multiple "denominators" and provide definitions

Report multiple "denominators" and provide definitions: Report N's (and effect sizes) "across a range of study participation [and use] thresholds" [1], e.g., N exposed, N consented, N used more than x times, N used more than y weeks, N participants "used" the intervention/comparator at specific pre-defined time points of interest (in absolute and relative numbers per group). Always clearly define "use" of the intervention.

subitem not at all important ○ ○ ○ ○ ◉ essential

Does your paper address subitem 16-i? *

Copy and paste relevant sections from the manuscript (include quotes in quotation marks "like this" to indicate direct quotes from your manuscript), or elaborate on this item by providing additional information not in the ms, or briefly explain why the item is not applicable/relevant for your study

"There was no significant (P>.016) difference between IG and CG with regard to acceptance, uptake, or adherence. Among the total sample, 9% (10/115) showed a low, 59% (68/115) a moderate, and 31% (36/115) a high level of acceptance, with an average sum score of 13.76 (SD 3.54). Figure 3 displays the levels of acceptance in both groups. The participants who applied for access to ACTonPain numbered 48 in IG and 50 in CG.
Note that 9% (5/57) and 10% (6/58) of participants in IG and CG, respectively, did not complete the survey and therefore did not indicate whether they wanted to receive the intervention. Then, 7% (4/57) and 3% (2/58) of participants in IG and CG, respectively, did not want to receive the intervention, and 84% (48/57) and 86% (50/58) of participants in IG and CG, respectively, signed up to receive the intervention. Four months after receiving access to ACTonPain, 65% (75/115) of the sample had logged in. This represents an uptake rate of 68% (38/57, IG) and 62% (36/58, CG). With regard to adherence, the participants completed 1.09 (SD 1.72) modules on average, that is, the average participant only completed the introduction module. The results showed that 5% (6/115) participants did not complete any modules after log-in and 3% (4/115) completed all the modules in the study. Hence, the treatment dropout rate was at 97% (111/115). Figure 4 presents the number of log-ins and completed modules in each group."

16-ii) Primary analysis should be intent-to-treat

Primary analysis should be intent-to-treat, secondary analyses could include comparing only "users", with the appropriate caveats that this is no longer a randomized sample (see 18-i).

subitem not at all important ○ ○ ○ ○ ◉ essential

Does your paper address subitem 16-ii?

Copy and paste relevant sections from the manuscript (include quotes in quotation marks "like this" to indicate direct quotes from your manuscript), or elaborate on this item by providing additional information not in the ms, or briefly explain why the item is not applicable/relevant for your study

"The descriptive statistics were based on nonimputed data, while all following analyses were conducted after multiple imputations with 20 imputations using the imputation algorithm implemented in SPSS (intention-to-treat analysis)."

17a) For each primary and secondary outcome, results for each group, and the estimated effect size and its precision (such as 95% confidence interval)

Does your paper address CONSORT subitem 17a? *

Copy and paste relevant sections from the manuscript (include quotes in quotation marks "like this" to indicate direct quotes from your manuscript), or elaborate on this item by providing additional information not in the ms, or briefly explain why the item is not applicable/relevant for your study

"Differences between IG and CG in all outcomes are summarized in Table 3."
"There was no significant (P>.016) difference between IG and CG with regard to acceptance, uptake, or adherence. "
"There was no significant (P>.05) difference between IG and CG with regard to performance expectancy, effort expectancy, social influence, facilitating conditions, or internet anxiety (Table 2)."

17a-i) Presentation of process outcomes such as metrics of use and intensity of use

In addition to primary/secondary (clinical) outcomes, the presentation of process outcomes such as metrics of use and intensity of use (dose, exposure) and their operational definitions is critical. This does not only refer to metrics of attrition (13-b) (often a binary variable), but also to more continuous exposure metrics such as "average session length". These must be accompanied by a technical description how a metric like a "session" is defined (e.g., timeout after idle time) [1] (report under item 6a).

subitem not at all important ○ ○ ○ ○ ◉ essential

Does your paper address subitem 17a-i?

Copy and paste relevant sections from the manuscript (include quotes in quotation marks "like this" to indicate direct quotes from your manuscript), or elaborate on this item by providing additional information not in the ms, or briefly explain why the item is not applicable/relevant for your study

Usage is one of the primary outcomes: "Note that 9% (5/57) and 10% (6/58) of participants in IG and CG, respectively, did not complete the survey and therefore did not indicate whether they wanted to receive the intervention. Then, 7% (4/57) and 3% (2/58) of participants in IG and CG, respectively, did not want to receive the intervention, and 84% (48/57) and 86% (50/58) of participants in IG and CG, respectively, signed up to receive the intervention. Four months after receiving access to ACTonPain, 65% (75/115) of the sample had logged in. This represents an uptake rate of 68% (38/57, IG) and 62% (36/58, CG). With regard to adherence, the participants completed 1.09 (SD 1.72) modules on average. That is, the average participant only completed the introduction module. The results showed that 5% (6/115) participants did not complete any modules after log-in and 3% (4/115) completed all the modules in the study. Hence, the treatment dropout rate was at 97% (111/115). Figure 4 presents the number of log-ins and completed modules in each group."

17b) For binary outcomes, presentation of both absolute and relative effect sizes is recommended

Does your paper address CONSORT subitem 17b? *

Copy and paste relevant sections from the manuscript (include quotes in quotation marks "like this" to indicate direct quotes from your manuscript), or elaborate on this item by providing additional information not in the ms, or briefly explain why the item is not applicable/relevant for your study

"Four months after receiving access to ACTonPain, 65% (75/115) of the sample had logged in. "

18) Results of any other analyses performed, including subgroup analyses and adjusted analyses, distinguishing pre-specified from exploratory

Does your paper address CONSORT subitem 18? *

Copy and paste relevant sections from the manuscript (include quotes in quotation marks "like this" to indicate direct quotes from your manuscript), or elaborate on this item by providing additional information not in the ms, or briefly explain why the item is not applicable/relevant for your study

"Since there were no group effects, we conducted the subgroup analyses with no group consideration in order to increase the power of the analyses. Participants with lower internet anxiety and higher anxiety symptoms showed significantly higher acceptance than their equivalent counterparts (Table 4). With regard to up-take rates, more participants with higher depressive symptoms (75%, 45/60) and acceptance (80%, 47/59) logged into the platform than those with lower depres-sive symptoms (55%, 30/55) and acceptance (50%, 28/56). We also found that participants with a higher level of acceptance completed more modules compared with participants with a lower level of acceptance (1.43 vs 0.72 modules). "

18-i) Subgroup analysis of comparing only users

A subgroup analysis of comparing only users is not uncommon in ehealth trials, but if done, it must be stressed that this is a self-selected sample and no longer an unbiased sample from a randomized trial (see 16-iii).

subitem not at all important ○ ○ ○ ○ ● essential

Does your paper address subitem 18-i?

Copy and paste relevant sections from the manuscript (include quotes in quotation marks "like this" to indicate direct quotes from your manuscript), or elaborate on this item by providing additional information not in the ms, or briefly explain why the item is not applicable/relevant for your study

"To examine potential subgroup differences (age, gender, education, pain duration and intensity, prior or present psychological intervention, internet usage and anxiety, and physical and emotional functioning) regarding acceptance, uptake, and adherence, exploratory analyses are provided (mean, SD, t-tests, and chi-square test). For this purpose, variables were dichotomized using defined cutoffs (gender, pain duration, education, and psychological intervention) or a median split (age, pain intensity, internet usage and anxiety, physical and emotional functioning, and level of acceptance regarding uptake and adherence). Note that the results of the subgroup analyses and analysis on secondary outcomes are exploratory and underpowered; adjusting for multiple testing would not be meaningful [75]."

19) All important harms or unintended effects in each group

(for specific guidance see CONSORT for harms)

Does your paper address CONSORT subitem 19? *

Copy and paste relevant sections from the manuscript (include quotes in quotation marks "like this" to indicate direct quotes from your manuscript), or elaborate on this item by providing additional information not in the ms, or briefly explain why the item is not applicable/relevant for your study

harms or unintended effects are not expected and not reported in an earlier study on the same intervention

19-i) Include privacy breaches, technical problems

Include privacy breaches, technical problems. This does not only include physical “harm” to participants, but also incidents such as perceived or real privacy breaches [1], technical problems, and other unexpected/unintended incidents. “Unintended effects” also includes unintended positive effects [2].

subitem not at all important ○ ○ ○ ○ ● essential

Does your paper address subitem 19-i?

Copy and paste relevant sections from the manuscript (include quotes in quotation marks "like this" to indicate direct quotes from your manuscript), or elaborate on this item by providing additional information not in the ms, or briefly explain why the item is not applicable/relevant for your study

no

19-ii) Include qualitative feedback from participants or observations from staff/researchers

Include qualitative feedback from participants or observations from staff/researchers, if available, on strengths and shortcomings of the application, especially if they point to unintended/unexpected effects or uses. This includes (if available) reasons for why people did or did not use the application as intended by the developers.

subitem not at all important ○ ○ ○ ○ ◉ essential

Does your paper address subitem 19-ii?

Copy and paste relevant sections from the manuscript (include quotes in quotation marks "like this" to indicate direct quotes from your manuscript), or elaborate on this item by providing additional information not in the ms, or briefly explain why the item is not applicable/relevant for your study

no

DISCUSSION

22) Interpretation consistent with results, balancing benefits and harms, and considering other relevant evidence

NPT: In addition, take into account the choice of the comparator, lack of or partial blinding, and unequal expertise of care providers or centers in each group

22-i) Restate study questions and summarize the answers suggested by the data, starting with primary outcomes and process outcomes (use)

Restate study questions and summarize the answers suggested by the data, starting with primary outcomes and process outcomes (use).

subitem not at all important ○ ○ ○ ○ ◉ essential

Does your paper address subitem 22-i? *

Copy and paste relevant sections from the manuscript (include quotes in quotation marks "like this" to indicate direct quotes from your manuscript), or elaborate on this item by providing additional information not in the ms, or briefly explain why the item is not applicable/relevant for your study

"Principal Findings
To the best of our knowledge, this study is the first to examine the impact of AFI on patients' acceptance, actual uptake, and adherence of an IMI. AFI consisted of a short informational video.
In this study, the average level of acceptance indicated a moderate to high acceptance in the sample (mean 13.76, SD 3.54) with no group differences between IG and CG. This acceptance level is higher than the levels examined in equivalent previous studies [42,46,47]. In these studies, acceptance levels in the intervention group after receiving IMI were at a mean of 11.42 (SD 4.28), 12.17 (SD 4.22), and 10.55 (SD 4.69) in samples of patients with depression [42], pain [47], and diabetes [46], respectively, in routine health care settings. The control groups in these studies displayed substantially lower levels of acceptance with M<10, indicating a low acceptance level on average. Contrary to previous studies, AFI in our study did not influence acceptance and its predictors, performance expectancy, effort expectancy, social influence, facilitating conditions, or internet anxiety. This might be due to the high baseline level of acceptance in the sample."

22-ii) Highlight unanswered new questions, suggest future research

Highlight unanswered new questions, suggest future research.

subitem not at all important ○ ○ ○ ○ ◉ essential

Does your paper address subitem 22-ii?

Copy and paste relevant sections from the manuscript (include quotes in quotation marks "like this" to indicate direct quotes from your manuscript), or elaborate on this item by providing additional information not in the ms, or briefly explain why the item is not applicable/relevant for your study

"Therefore, future research should be conducted within naturalistic settings with more representative samples. Further, strategies to increase adherence in IMIs need to be developed involving IMI users, developers, and providers."

20) Trial limitations, addressing sources of potential bias, imprecision, and, if relevant, multiplicity of analyses

20-i) Typical limitations in ehealth trials

Typical limitations in ehealth trials: Participants in ehealth trials are rarely blinded. Ehealth trials often look at a multiplicity of outcomes, increasing risk for a Type I error. Discuss biases due to non-use of the intervention/usability issues, biases through informed consent procedures, unexpected events.

subitem not at all important ○ ○ ○ ○ ◉ essential

Does your paper address subitem 20-i? *

Copy and paste relevant sections from the manuscript (include quotes in quotation marks "like this" to indicate direct quotes from your manuscript), or elaborate on this item by providing additional information not in the ms, or briefly explain why the item is not applicable/relevant for your study

"Limitations
Several limitations in this study are noteworthy. First, the recruiting strategy might have influenced the way the participants filled out the survey, and their answers might have been more socially desirable. Consequently, the results on acceptance and uptake might not be representative for the population of patients with chronic pain, but they are likely to be representative for the population of patients with chronic pain in previous efficacy trials on IMIs for chronic pain. Hence, this study provides information on participants' acceptance in efficacy studies that can be useful for the interpretation of their respective results. This is especially the case regarding their generalizability to routine clinical practice given that most of these studies are conducted under ideal circumstances with highly specified inclusion and exclusion criteria [76].
In connection with the abovementioned lack of implementation facilitating factors in our AFI, a further limitation of this study is that it is only based on the UTAUT model. The UTAUT model and other equivalent models on the acceptance of IMIs as evaluated in a previous study [74], as well as in some empirical studies [27,53], suggest a relationship between acceptance and IMI use but might not consider sus-tained use, which is required in IMIs. Therefore, the findings of our study indicate that adherence facilitating factors are crucial even when acceptance is high. Hence, future research is needed to test interventions aimed at increasing adherence. HAPA can serve as an intervention model.
Finally, the reliability of the acceptance scale was relatively low at.71 compared with previous studies (Cronbach alpha ranged from.84 [42] to.87 [46,47]). Howev-er, the Cronbach alpha in this study is still in an acceptable range, especially as the scale consists of only 4 items [87]."

21) Generalisability (external validity, applicability) of the trial findings

NPT: External validity of the trial findings according to the intervention, comparators, patients, and care providers or centers involved in the trial

21-i) Generalizability to other populations

Generalizability to other populations: In particular, discuss generalizability to a general Internet population, outside of a RCT setting, and general patient population, including applicability of the study results for other organizations

subitem not at all important ○ ○ ○ ○ ◉ essential

Does your paper address subitem 21-i?

Copy and paste relevant sections from the manuscript (include quotes in quotation marks "like this" to indicate direct quotes from your manuscript), or elaborate on this item by providing additional information not in the ms, or briefly explain why the item is not applicable/relevant for your study

"The comparatively high acceptance in both groups of this study is potentially due to selective sampling. We recruited the participants from a pool of persons who had already expressed interest in participating in a previous study on ACTonPain. After the end of recruitment for the main study, we invited all persons who were not randomized in the study to participate in this study and to receive ACTonPain as an incentive after completion of the survey. Hence, the participants in this study expressed their interest for ACTonPain twice. Therefore, the level of acceptance most likely reflects the acceptance and uptake in many IMI efficacy studies consist-ing of a population that is considerably more interested and open to IMIs than the general population [76]."

21-ii) Discuss if there were elements in the RCT that would be different in a routine application setting

Discuss if there were elements in the RCT that would be different in a routine application setting (e.g., prompts/reminders, more human involvement, training sessions or other co-interventions) and what impact the omission of these elements could have on use, adoption, or outcomes if the intervention is applied outside of a RCT setting.

subitem not at all important ○ ○ ○ ○ ◉ essential

Does your paper address subitem 21-ii?

Copy and paste relevant sections from the manuscript (include quotes in quotation marks "like this" to indicate direct quotes from your manuscript), or elaborate on this item by providing additional information not in the ms, or briefly explain why the item is not applicable/relevant for your study

"The comparatively high acceptance in both groups of this study is potentially due to selective sampling. We recruited the participants from a pool of persons who had already expressed interest in participating in a previous study on ACTonPain. After the end of recruitment for the main study, we invited all persons who were not randomized in the study to participate in this study and to receive ACTonPain as an incentive after completion of the survey. Hence, the participants in this study expressed their interest for ACTonPain twice. Therefore, the level of acceptance most likely reflects the acceptance and uptake in many IMI efficacy studies consist-ing of a population that is considerably more interested and open to IMIs than the general population [76]. Therefore, our previous work on acceptance in the general population [42,46,47] might give us a more realistic estimate of acceptance. By comparing the acceptance rates throughout the studies, this study quantifies how acceptance and uptake rates can differ between populations in efficacy studies and routine health care settings."

OTHER INFORMATION

23) Registration number and name of trial registry

Does your paper address CONSORT subitem 23? *

Copy and paste relevant sections from the manuscript (include quotes in quotation marks "like this" to indicate direct quotes from your manuscript), or elaborate on this item by providing additional information not in the ms, or briefly explain why the item is not applicable/relevant for your study

"Trial Registration: Is study is linked to an outcome evaluation study with the German Clinical Trial Registration (DRKS): DRKS00006183"

24) Where the full trial protocol can be accessed, if available

Does your paper address CONSORT subitem 24? *

Cite a Multimedia Appendix, other reference, or copy and paste relevant sections from the manuscript (include quotes in quotation marks "like this" to indicate direct quotes from your manuscript), or elaborate on this item by providing additional information not in the ms, or briefly explain why the item is not applicable/relevant for your study

not applicable

25) Sources of funding and other support (such as supply of drugs), role of funders

Does your paper address CONSORT subitem 25? *

Copy and paste relevant sections from the manuscript (include quotes in quotation marks "like this" to indicate direct quotes from your manuscript), or elaborate on this item by providing additional information not in the ms, or briefly explain why the item is not applicable/relevant for your study

"This work was supported by a postdoc fellowship of the German Academic Exchange Service (DAAD)"

X27) Conflicts of Interest (not a CONSORT item)

X27-i) State the relation of the study team towards the system being evaluated

In addition to the usual declaration of interests (financial or otherwise), also state the relation of the study team towards the system being evaluated, i.e., state if the authors/evaluators are distinct from or identical with the developers/sponsors of the intervention.

subitem not at all important ○ ○ ○ ○ ◉ essential

Does your paper address subitem X27-i?

Copy and paste relevant sections from the manuscript (include quotes in quotation marks "like this" to indicate direct quotes from your manuscript), or elaborate on this item by providing additional information not in the ms, or briefly explain why the item is not applicable/relevant for your study

"None declared. "

About the CONSORT EHEALTH checklist

As a result of using this checklist, did you make changes in your manuscript? *

- ○ yes, major changes
- ◉ yes, minor changes
- ○ no

What were the most important changes you made as a result of using this checklist?

Meine Antwort

How much time did you spend on going through the checklist INCLUDING making changes in your manuscript *

4 h

As a result of using this checklist, do you think your manuscript has improved? *

- (x) yes
- ( ) no
- ( ) Sonstiges:

Would you like to become involved in the CONSORT EHEALTH group?

This would involve for example becoming involved in participating in a workshop and writing an "Explanation and Elaboration" document

- (x) yes
- ( ) no
- ( ) Sonstiges:

Any other comments or questions on CONSORT EHEALTH

Meine Antwort

STOP - Save this form as PDF before you click submit

To generate a record that you filled in this form, we recommend to generate a PDF of this page (on a Mac, simply select "print" and then select "print as PDF") before you submit it.

When you submit your (revised) paper to JMIR, please upload the PDF as supplementary file.

Don't worry if some text in the textboxes is cut off, as we still have the complete information in our database. Thank you!

Final step: Click submit !

Click submit so we have your answers in our database!

SENDEN

Geben Sie niemals Passwörter über Google Formulare weiter.

Dieser Inhalt wurde nicht von Google erstellt und wird von Google auch nicht unterstützt. Missbrauch melden - Nutzungsbedingungen - Zusätzliche Bestimmungen

Google Formulare
